# Next-generation ARIA care pathways for rhinitis and asthma: a model for multimorbid chronic diseases

**DOI:** 10.1186/s13601-019-0279-2

**Published:** 2019-09-09

**Authors:** J. Jean Bousquet, Holger J. Schünemann, Alkis Togias, Marina Erhola, Peter W. Hellings, Torsten Zuberbier, Ioana Agache, Ignacio J. Ansotegui, Josep M. Anto, Claus Bachert, Sven Becker, Martin Bedolla-Barajas, Michael Bewick, Sinthia Bosnic-Anticevich, Isabelle Bosse, Louis P. Boulet, Jean Marc Bourrez, Guy Brusselle, Niels Chavannes, Elisio Costa, Alvaro A. Cruz, Wienczyslawa Czarlewski, Wytske J. Fokkens, Joao A. Fonseca, Mina Gaga, Tari Haahtela, Maddalena Illario, Ludger Klimek, Piotr Kuna, Violeta Kvedariene, L. T. T. Le, Desiree Larenas-Linnemann, Daniel Laune, Olga M. Lourenço, Enrica Menditto, Joaquin Mullol, Yashitaka Okamoto, Nikos Papadopoulos, Nhân Pham-Thi, Robert Picard, Hilary Pinnock, Nicolas Roche, Regina E. Roller-Wirnsberger, Christine Rolland, Boleslaw Samolinski, Aziz Sheikh, Sanna Toppila-Salmi, Ioanna Tsiligianni, Arunas Valiulis, Erkka Valovirta, Tuula Vasankari, Maria-Teresa Ventura, Samantha Walker, Sian Williams, Cezmi A. Akdis, Isabella Annesi-Maesano, Sylvie Arnavielhe, Xavier Basagana, Eric Bateman, Anna Bedbrook, K. S. Bennoor, Samuel Benveniste, Karl C. Bergmann, Slawomir Bialek, Nils Billo, Carsten Bindslev-Jensen, Leif Bjermer, Hubert Blain, Mateo Bonini, Philippe Bonniaud, Jacques Bouchard, Vitalis Briedis, Christofer E. Brightling, Jan Brozek, Roland Buhl, Roland Buonaiuto, Giorgo W. Canonica, Victoria Cardona, Ana M. Carriazo, Warner Carr, Christine Cartier, Thomas Casale, Lorenzo Cecchi, Alfonso M. Cepeda Sarabia, Eka Chkhartishvili, Derek K. Chu, Cemal Cingi, Elaine Colgan, Jaime Correia de Sousa, Anne Lise Courbis, Adnan Custovic, Biljana Cvetkosvki, Gennaro D’Amato, Jane da Silva, Carina Dantas, Dejand Dokic, Yves Dauvilliers, Antoni Dedeu, Giulia De Feo, Philippe Devillier, Stefania Di Capua, Marc Dykewickz, Ruta Dubakiene, Motohiro Ebisawa, Yaya El-Gamal, Esben Eller, Regina Emuzyte, John Farrell, Antjie Fink-Wagner, Alessandro Fiocchi, Jean F. Fontaine, Bilun Gemicioğlu, Peter Schmid-Grendelmeir, Amiran Gamkrelidze, Judith Garcia-Aymerich, Maximiliano Gomez, Sandra González Diaz, Maia Gotua, Nick A. Guldemond, Maria-Antonieta Guzmán, Jawad Hajjam, John O’B Hourihane, Marc Humbert, Guido Iaccarino, Despo Ierodiakonou, Maddalena Illario, Juan C. Ivancevich, Guy Joos, Ki-Suck Jung, Marek Jutel, Igor Kaidashev, Omer Kalayci, Przemyslaw Kardas, Thomas Keil, Mussa Khaitov, Nikolai Khaltaev, Jorg Kleine-Tebbe, Marek L. Kowalski, Vicky Kritikos, Inger Kull, Lisa Leonardini, Philip Lieberman, Brian Lipworth, Karin C. Lodrup Carlsen, Claudia C. Loureiro, Renaud Louis, Alpana Mair, Gert Marien, Bassam Mahboub, Joao Malva, Patrick Manning, Esteban De Manuel Keenoy, Gailen D. Marshall, Mohamed R. Masjedi, Jorge F. Maspero, Eve Mathieu-Dupas, Poalo M. Matricardi, Eric Melén, Elisabete Melo-Gomes, Eli O. Meltzer, Enrica Menditto, Jacques Mercier, Neven Miculinic, Florin Mihaltan, Branislava Milenkovic, Giuliana Moda, Maria-Dolores Mogica-Martinez, Yousser Mohammad, Steve Montefort, Ricardo Monti, Mario Morais-Almeida, Ralf Mösges, Lars Münter, Antonella Muraro, Ruth Murray, Robert Naclerio, Luigi Napoli, Leila Namazova-Baranova, Hugo Neffen, Kristoff Nekam, Angelo Neou, Enrico Novellino, Dieudonné Nyembue, Robin O’Hehir, Ken Ohta, Kimi Okubo, Gabrielle Onorato, Solange Ouedraogo, Isabella Pali-Schöll, Susanna Palkonen, Peter Panzner, Hae-Sim Park, Jean-Louis Pépin, Ana-Maria Pereira, Oliver Pfaar, Ema Paulino, Jim Phillips, Robert Picard, Davor Plavec, Ted A. Popov, Fabienne Portejoie, David Price, Emmanuel P. Prokopakis, Benoit Pugin, Filip Raciborski, Rojin Rajabian-Söderlund, Sietze Reitsma, Xavier Rodo, Antonino Romano, Nelson Rosario, Menahenm Rottem, Dermot Ryan, Johanna Salimäki, Mario M. Sanchez-Borges, Juan-Carlos Sisul, Dirceu Solé, David Somekh, Talant Sooronbaev, Milan Sova, Otto Spranger, Cristina Stellato, Rafael Stelmach, Charlotte Suppli Ulrik, Michel Thibaudon, Teresa To, Ana Todo-Bom, Peter V. Tomazic, Antonio A. Valero, Rudolph Valenta, Marylin Valentin-Rostan, Rianne van der Kleij, Olivier Vandenplas, Giorgio Vezzani, Frédéric Viart, Giovanni Viegi, Dana Wallace, Martin Wagenmann, De Y. Wang, Susan Waserman, Magnus Wickman, Dennis M. Williams, Gary Wong, Piotr Wroczynski, Panayiotis K. Yiallouros, Arzu Yorgancioglu, Osman M. Yusuf, Heahter J. Zar, Stéphane Zeng, Mario Zernotti, Luo Zhang, Nan S. Zhong, Mihaela Zidarn, the ARIA Study Group, the MASK Study Group, J. Bousquet, P. W. Hellings, W. Aberer, I. Agache, C. A. Akdis, M. Akdis, M. R. Aliberti, R. Almeida, F. Amat, R. Angles, I. Annesi-Maesano, I. J. Ansotegui, J. M. Anto, S. Arnavielle, E. Asayag, A. Asarnoj, H. Arshad, F. Avolio, E. Bacci, C. Bachert, I. Baiardini, C. Barbara, M. Barbagallo, I. Baroni, B. A. Barreto, X. Basagana, E. D. Bateman, M. Bedolla-Barajas, A. Bedbrook, M. Bewick, B. Beghé, E. H. Bel, K. C. Bergmann, K. S. Bennoor, M. Benson, L. Bertorello, A. Z. Białoszewski, T. Bieber, S. Bialek, C. Bindslev-Jensen, L. Bjermer, H. Blain, F. Blasi, A. Blua, M. Bochenska Marciniak, I. Bogus-Buczynska, A. L. Boner, M. Bonini, S. Bonini, C. S. Bosnic-Anticevich, I. Bosse, J. Bouchard, L. P. Boulet, R. Bourret, P. J. Bousquet, F. Braido, V. Briedis, C. E. Brightling, J. Brozek, C. Bucca, R. Buhl, R. Buonaiuto, C. Panaitescu, M. T. Burguete Cabañas, E. Burte, A. Bush, F. Caballero-Fonseca, D. Caillaud, D. Caimmi, M. A. Calderon, P. A. M. Camargos, T. Camuzat, G. Canfora, G. W. Canonica, V. Cardona, K. H. Carlsen, P. Carreiro-Martins, A. M. Carriazo, W. Carr, C. Cartier, T. Casale, G. Castellano, L. Cecchi, A. M. Cepeda, N. H. Chavannes, Y. Chen, R. Chiron, T. Chivato, E. Chkhartishvili, A. G. Chuchalin, K. F. Chung, M. M. Ciaravolo, A. Ciceran, C. Cingi, G. Ciprandi, A. C. Carvalho Coehlo, L. Colas, E. Colgan, J. Coll, D. Conforti, J. Correia de Sousa, R. M. Cortés-Grimaldo, F. Corti, E. Costa, M. C. Costa-Dominguez, A. L. Courbis, L. Cox, M. Crescenzo, A. A. Cruz, A. Custovic, W. Czarlewski, S. E. Dahlen, G. D’Amato, C. Dario, J. da Silva, Y. Dauvilliers, U. Darsow, F. De Blay, G. De Carlo, T. Dedeu, M. de Fátima Emerson, G. De Feo, G. De Vries, B. De Martino, N. P. Motta Rubina, D. Deleanu, P. Demoly, J. A. Denburg, P. Devillier, S. Di Capua Ercolano, N. Di Carluccio, A. Didier, D. Dokic, M. G. Dominguez-Silva, H. Douagui, G. Dray, R. Dubakiene, S. R. Durham, G. Du Toit, M. S. Dykewicz, Y. El-Gamal, P. Eklund, E. Eller, R. Emuzyte, J. Farrell, A. Farsi, J. Ferreira de Mello, J. Ferrero, A. Fink-Wagner, A. Fiocchi, W. J. Fokkens, J. A. Fonseca, J. F. Fontaine, S. Forti, J. M. Fuentes-Perez, J. L. Gálvez-Romero, A. Gamkrelidze, J. Garcia-Aymerich, C. Y. García-Cobas, M. H. Garcia-Cruz, B. Gemicioğlu, S. Genova, G. Christoff, J. E. Gereda, R. Gerth van Wijk, R. M. Gomez, J. Gómez-Vera, S. González Diaz, M. Gotua, I. Grisle, M. Guidacci, N. A. Guldemond, Z. Gutter, M. A. Guzmán, T. Haahtela, J. Hajjam, L. Hernández, J. O’B Hourihane, Y. R. Huerta-Villalobos, M. Humbert, G. Iaccarino, M. Illario, Z. Ispayeva, J. C. Ivancevich, E. J. Jares, E. Jassem, S. L. Johnston, G. Joos, K. S. Jung, J. Just, M. Jutel, I. Kaidashev, O. Kalayci, A. F. Kalyoncu, J. Karjalainen, P. Kardas, T. Keil, P. K. Keith, M. Khaitov, N. Khaltaev, J. Kleine-Tebbe, L. Klimek, M. L. Kowalski, M. Kuitunen, I. Kull, P. Kuna, M. Kupczyk, V. Kvedariene, E. Krzych-Fałta, P. Lacwik, D. Larenas-Linnemann, D. Laune, D. Lauri, J. Lavrut, L. T. T. Le, M. Lessa, G. Levato, J. Li, P. Lieberman, A. Lipiec, B. Lipworth, K. C. Lodrup Carlsen, R. Louis, O. Lourenço, J. A. Luna-Pech, A. Magnan, B. Mahboub, D. Maier, A. Mair, I. Majer, J. Malva, E. Mandajieva, P. Manning, E. De Manuel Keenoy, G. D. Marshall, M. R. Masjedi, J. F. Maspero, E. Mathieu-Dupas, J. J. Matta Campos, A. L. Matos, M. Maurer, S. Mavale-Manuel, O. Mayora, M. A. Medina-Avalos, E. Melén, E. Melo-Gomes, E. O. Meltzer, E. Menditto, J. Mercier, N. Miculinic, F. Mihaltan, B. Milenkovic, G. Moda, M. D. Mogica-Martinez, Y. Mohammad, I. Momas, S. Montefort, R. Monti, D. Mora Bogado, M. Morais-Almeida, F. F. Morato-Castro, R. Mösges, A. Mota-Pinto, P. Moura Santo, J. Mullol, L. Münter, A. Muraro, R. Murray, R. Naclerio, R. Nadif, M. Nalin, L. Napoli, L. Namazova-Baranova, H. Neffen, V. Niedeberger, K. Nekam, A. Neou, A. Nieto, L. Nogueira-Silva, M. Nogues, E. Novellino, T. D. Nyembue, R. E. O’Hehir, C. Odzhakova, K. Ohta, Y. Okamoto, K. Okubo, G. L. Onorato, M. Ortega Cisneros, S. Ouedraogo, I. Pali-Schöll, S. Palkonen, P. Panzner, N. G. Papadopoulos, H. S. Park, A. Papi, G. Passalacqua, E. Paulino, R. Pawankar, S. Pedersen, J. L. Pépin, A. M. Pereira, M. Persico, O. Pfaar, J. Phillips, R. Picard, B. Pigearias, I. Pin, C. Pitsios, D. Plavec, W. Pohl, T. A. Popov, F. Portejoie, P. Potter, A. C. Pozzi, D. Price, E. P. Prokopakis, R. Puy, B. Pugin, R. E. Pulido Ross, M. Przemecka, K. F. Rabe, F. Raciborski, R. Rajabian-Soderlund, S. Reitsma, I. Ribeirinho, J. Rimmer, D. Rivero-Yeverino, J. A. Rizzo, M. C. Rizzo, C. Robalo-Cordeiro, F. Rodenas, X. Rodo, M. Rodriguez Gonzalez, L. Rodriguez-Mañas, C. Rolland, S. Rodrigues Valle, M. Roman Rodriguez, A. Romano, E. Rodriguez-Zagal, G. Rolla, R. E. Roller-Wirnsberger, M. Romano, J. Rosado-Pinto, N. Rosario, M. Rottem, D. Ryan, H. Sagara, J. Salimäki, B. Samolinski, M. Sanchez-Borges, J. Sastre-Dominguez, G. K. Scadding, H. J. Schunemann, N. Scichilone, P. Schmid-Grendelmeier, F. S. Serpa, S. Shamai, A. Sheikh, M. Sierra, F. E. R. Simons, V. Siroux, J. C. Sisul, I. Skrindo, D. Solé, D. Somekh, M. Sondermann, T. Sooronbaev, M. Sova, M. Sorensen, M. Sorlini, O. Spranger, C. Stellato, R. Stelmach, R. Stukas, J. Sunyer, J. Strozek, A. Szylling, J. N. Tebyriçá, M. Thibaudon, T. To, A. Todo-Bom, P. V. Tomazic, S. Toppila-Salmi, U. Trama, M. Triggiani, C. Suppli Ulrik, M. Urrutia-Pereira, R. Valenta, A. Valero, A. Valiulis, E. Valovirta, M. van Eerd, E. van Ganse, M. van Hage, O. Vandenplas, M. T. Ventura, G. Vezzani, T. Vasankari, A. Vatrella, M. T. Verissimo, F. Viart, G. Viegi, D. Vicheva, T. Vontetsianos, M. Wagenmann, S. Walker, D. Wallace, D. Y. Wang, S. Waserman, T. Werfel, M. Westman, M. Wickman, D. M. Williams, S. Williams, N. Wilson, J. Wright, P. Wroczynski, P. Yakovliev, B. P. Yawn, P. K. Yiallouros, A. Yorgancioglu, O. M. Yusuf, H. J. Zar, L. Zhang, N. Zhong, M. E. Zernotti, I. Zhanat, M. Zidarn, T. Zuberbier, C. Zubrinich, A. Zurkuhlen

**Affiliations:** 1 MACVIA-France, Fondation Partenariale FMC VIA-LR, CHU, 34295 Montpellier Cedex 5, France; 2 INSERM U 1168, VIMA: Ageing and Chronic Diseases Epidemiological and Public Health Approaches, Villejuif Université Versailles St-Quentin-en-Yvelines, UMR-S 1168, Montigny Le Bretonneux, France; 3 European Forum for Research and Education in Allergy and Airway Diseases (EUFOREA), Brussels, Belgium; 4 Charité-Universitätsmedizin Berlin, Corporate Member of Freie Universität Berlin, Humboldt-Uniersität zu Berlin, Berlin, Germany; 5 Department of Dermatology and Allergy Member of GA2LEN, Comprehensive Allergy-Centre, Berlin Institute of Health, Berlin, Germany; 6 Department of Clinical Epidemiology and Biostatistics, McMaster University, Hamilton, ON Canada; 7 Division of Allergy, Immunology, and Transplantation (DAIT), National Institute of Allergy and Infectious Diseases, NIH, Bethesda, USA; 8 National Institute for Health and Welfare, Helsinki, Finland; 9 Dept of Otorhinolaryngology, Univ Hospitals Leuven, Louvain, Belgium; 10 Academic Medical Center, Univ of Amsterdam, Amsterdam, The Netherlands; 11 Faculty of Medicine, Transylvania University, Brasov, Romania; 12 Department of Allergy and Immunology, Hospital Quirónsalud Bizkaia, Erandio, Spain; 13 ISGlobAL, Centre for Research in Environmental Epidemiology (CREAL), Barcelona, Spain; 14 IMIM (Hospital del Mar Research Institute), Barcelona, Spain; 15 CIBER Epidemiología y Salud Pública (CIBERESP), Barcelona, Spain; 16 Universitat Pompeu Fabra (UPF), Barcelona, Spain; 17 Upper Airways Research Laboratory, ENT Dept, Ghent University Hospital, Ghent, Belgium; 18 Dept of Otolaryngology, Head and Neck Surgery, University of Mainz, Mainz, Germany; 19 Hospital Civil de Guadalajara Dr Juan I Menchaca, Guadalarara, Mexico; 20 iQ4U Consultants Ltd, London, UK; 21 Woolcock Institute of Medical Research, University of Sydney, Sydney, Australia; 22 Woolcock Emphysema Centre, Sydney, Australia; 23 Sydney Local Health District, Glebe, NSW Australia; 24 La Rochelle, France; 25 Quebec Heart and Lung Institute, Laval University, Quebec City, QC Canada; 26 EIT Health France, Paris, France; 27 Dept of Respiratory Medicine, Ghent University Hospital, Ghent, Belgium; 28 Department of Public Health and Primary Care, Leiden University Medical Center, Leiden, The Netherlands; 29 UCIBIO, REQUINTE, Faculty of Pharmacy and Competence Center on Active and Healthy Ageing of University of Porto (Porto4Ageing), Porto, Portugal; 30 ProAR–Nucleo de Excelencia em Asma, Federal University of Bahia, Bahia, Brazil; 31 WHO GARD Planning Group, Salvador, Brazil; 32 Medical Consulting Czarlewski, Levallois, France; 33 Department of Otorhinolaryngology, Amsterdam University Medical Centres, AMC, Amsterdam, The Netherlands; 34 CINTESIS, Center for Research in Health Technology and Information Systems, Faculdade de Medicina da Universidade do Porto, Porto, Portugal; 35 Medida, Lda Porto, Portugal; 36 ERS President 2017-2018, Athens Chest Hospital, 7th Resp Med Dept and Asthma Center, Athens, Greece; 37 Skin and Allergy Hospital, Helsinki University Hospital and University of Helsinki, Helsinki, Finland; 38 Division for Health Innovation, Campania Region and Federico II University Hospital Naples (R &D and DISMET), Naples, Italy; 39 Center for Rhinology and Allergology, Wiesbaden, Germany; 40 Division of Internal Medicine, Asthma and Allergy, Barlicki University Hospital, Medical University of Lodz, Łódź, Poland; 41 Faculty of Medicine, Vilnius University, Vilnius, Lithuania; 42 University of Medicine and Pharmacy, Hochiminh City, Vietnam; 43 Center of Excellence in Asthma and Allergy, Médica Sur Clinical Foundation and Hospital, México City, Mexico; 44 KYomed INNOV, Montpellier, France; 45 Faculty of Health Sciences and CICS–UBI, Health Sciences Research Centre, University of Beira Interior, Covilhã, Portugal; 46 CIRFF, Federico II University, Naples, Italy; 47 Rhinology Unit & Smell Clinic, ENT Department, Hospital Clínic, Barcelona, Spain; 48 Clinical & Experimental Respiratory Immunoallergy, IDIBAPS, CIBERES, University of Barcelona, Barcelona, Spain; 49 Dept of Otorhinolaryngology, Chiba University Hospital, Chiba, Japan; 50 Division of Infection, Immunity & Respiratory Medicine, Royal Manchester Children’s Hospital, University of Manchester, Manchester, UK; 51 Allergy Department, 2nd Pediatric Clinic, Athens General Children’s Hospital “P&A Kyriakou”, University of Athens, Athens, Greece; 52 Allergy Department, Pasteur Institute, Paris, France; 53 Conseil Général de l’Economie Ministère de l’Economie, de l’Industrie et du Numérique, Paris, France; 54 The Usher Institute of Population Health Sciences and Informatics, The University of Edinburgh, Edinburgh, UK; 55 Pneumologie et Soins Intensifs Respiratoires, Hôpitaux Universitaires Paris, Centre Hôpital Cochin, Paris, France; 56 Department of Internal Medicine, Medical University of Graz, Graz, Austria; 57 Association Asthme et Allergie, Paris, France; 58 Department of Prevention of Environmental Hazards and Allergology, Medical University of Warsaw, Warsaw, Poland; 59 Health Planning Unit, Department of Social Medicine, Faculty of Medicine, University of Crete, Crete, Greece; 60 International Primary Care Respiratory Group IPCRG, Aberdeen, Scotland UK; 61 Institute of Clinical Medicine & Institute of Health Sciences, Vilnius University Faculty of Medicine, Vilnius, Lithuania; 62 Department of Lung Diseases and Clinical Immunology, University of Turku and Terveystalo Allergy Clinic, Turku, Finland; 63 FILHA, Finnish Lung Association, Helsinki, Finland; 64 Unit of Geriatric Immunoallergology, University of Bari Medical School, Bari, Italy; 65 Asthma UK, Mansell Street, London, UK; 66 Swiss Institute of Allergy and Asthma Research (SIAF), University of Zurich, Davos, Switzerland; 67 Epidemiology of Allergic and Respiratory Diseases, Department Institute Pierre Louis of Epidemiology and Public Health, INSERM and Sorbonne Université, Medical School Saint Antoine, Paris, France; 68 Department of Medicine, University of Cape Town, Cape Town, South Africa; 69 Dept of Respiratory Medicine, National Institute of Diseases of the Chest and Hospital, Dhaka, Bangladesh; 70 National Center of Expertise in Cognitive Stimulation (CEN STIMCO), Broca Hospital, Paris, France; 71 Department of Biochemistry and Clinical Chemistry, Faculty of Pharmacy with the Division of Laboratory Medicine, Warsaw Medical University, Warsaw, Poland; 72 Global Alliance against Chronic Respiratory Diseases (WHO GARD), Joensuu, Finland; 73 Department of Dermatology and Allergy Centre, Odense University Hospital, Odense Research Center for Anaphylaxis (ORCA), Odense, Denmark; 74 Termofischer Scientific, Uppsala, Sweden; 75 Department of Respiratory Medicine and Allergology, University Hospital, Lund, Sweden; 76 Department of Geriatrics, Montpellier University hospital, Montpellier, France; 77 EA 2991 Euromov, University Montpellier, Montpellier, France; 78 UOC Pneumologia, Istituto di Medicina Interna, F Policlinico Gemelli IRCCS, Università Cattolica del Sacro Cuore, Rome, Italy; 79 National Heart and Lung Institute, Royal Brompton Hospital & Imperial College, London, UK; 80 CHU, Dijon, France; 81 Clinical Medicine, Laval’s University, Quebec City, Canada; 82 Medicine Department, Hôpital de la Malbaie, Quebec city, QC Canada; 83 Department of Clinical Pharmacy of Lithuanian, University of Health, Kaunas, Lithuania; 84 Institute of Lung Health, Respiratory Biomedical Unit, University Hospitals of Leicester NHS Trust, Leicestershire, UK; 85 Department of Infection, Immunity and Inflammation, University of Leicester, Leicester, UK; 86 Universitätsmedizin der Johannes Gutenberg-Universität Mainz, Mainz, Germany; 87 Municipality Pharmacy, Sarno, Italy; 88 Personalized Medicine Clinic Asthma & Allergy, Humanitas University, Humanitas Research Hospital, Rozzano, Milan, Italy; 89 Allergy Section, Department of Internal Medicine, Hospital Vall d’Hebron & ARADyAL Research Network, Barcelona, Spain; 90 Regional Ministry of Health of Andalusia, Seville, Spain; 91 Allergy and Asthma Associates of Southern California, Mission Viejo, CA USA; 92 ASA-Advanced Solutions Accelerator, Clapiers, France; 93 Division of Allergy/Immunology, University of South Florida, Tampa, FL USA; 94 SOS Allergology and Clinical Immunology, USL Toscana Centro, Prato, Italy; 95 Allergy and Immunology Laboratory, Metropolitan University, Simon Bolivar University, Barranquilla, Colombia; 96 SLaai, Sociedad Latinoamericana de Allergia, Asma e Immunologia, Barranquilla, Colombia; 97 Chachava Clinic, David Tvildiani Medical University-AIETI Medical School, Grigol Robakidze University, Tbilisi, Georgia; 98 Medical Faculty, ENT Department, Eskisehir Osmangazi University, Eskisehir, Turkey; 99 Department of Health, Social Services and Public Safety, Belfast, Northern Ireland UK; 100 Life and Health Sciences Research Institute (ICVS), School of Medicine, University of Minho, Braga, Portugal; 101 PT Government Associate Laboratory, ICVS/3B’s, Braga/Guimarães, Portugal; 102 Ecole des Mines, Alès, France; 103 Centre for Respiratory Medicine and Allergy, Institute of Inflammation and Repair, University of Manchester and University Hospital of South Manchester, Manchester, UK; 104 Division of Respiratory and Allergic Diseases, Department of Respiratory Diseases, High Specialty Hospital A.Cardarelli, Naples, Italy; 105 Allergy Service, University Hospital of Federal University of Santa Catarina (HU-UFSC), Florianópolis, Brazil; 106 Cáritas Diocesana de Coimbra, Coimbra, Portugal; 107 Ageing@Coimbra EIP-AHA Reference Site, Coimbra, Portugal; 108 Medical Faculty Skopje, University Clinic of Pulmonology and Allergy, Skopje, Republic of Macedonia; 109 Sleep Unit, Department of Neurology, Hôpital Gui-de-Chauliac Montpellier, Inserm U1061, Montpellier, France; 110 AQuAS, Barcelna, Spain; 111 EUREGHA, European Regional and Local Health Association, Brussels, Belgium; 112 Department of Medicine, Surgery and Dentistry “Scuola Medica Salernitana”, University of Salerno, Salerno, Italy; 113 UPRES EA220, Pôle des Maladies des Voies Respiratoires, Hôpital Foch, Université Paris-Saclay, Suresnes, France; 114 Farmacie Dei Golfi Group, Massa Lubrense, Italy; 115 Section of Allergy and Immunology, Saint Louis University School of Medicine, Saint Louis, MO USA; 116 Clinic of Infectious, Chest Diseases, Dermatology and Allergology, Vilnius University, Vilnius, Lithuania; 117 Clinical Reserch Center for Allergy and Rheumatology, Sagamihara National Hospital, Sagamihara, Japan; 118 Pediatric Allergy and Immunology Unit, Children’s hospital, Ain Shams University, Cairo, Egypt; 119 Clinic of Children’s Diseases, Faculty of Medicine, Vilnius University, Vilnius, Lithuania; 120 Global Allergy and Asthma Platform GAAPP, Vienna, Austria; 121 Division of Allergy, Department of Pediatric Medicine, The Bambino Gesù Children’s Research Hospital Holy See, Rome, Italy; 122 Reims, France; 123 Department of Pulmonary Diseases, Istanbul University-Cerrahpasa, Cerrahpasa Faculty of Medicine, Istambul, Turkey; 124 Allergy Unit, Department of Dermatology, University Hospital of Zurich, Zurich, Switzerland; 125 National Center for Disease Control and Public Health of Georgia, Tbilisi, Georgia; 126 Allergy & Asthma Unit, Hospital San Bernardo Salta, Salta, Argentina; 127 Universidad Autónoma de Nuevo León, San Nicolás de los Garza, Mexico; 128 Center of Allergy and Immunology, Georgian Association of Allergology and Clinical Immunology, Tbilisi, Georgia; 129 Institute of Health Policy and Management iBMG, Erasmus University, Rotterdam, The Netherlands; 130 Immunology and Allergy Division, Clinical Hospital, University of Chile, Santiago, Chile; 131 Centich: Centre d’Expertise National des Technologies de l’Information et de la Communication pour l’Autonomie, Gérontopôle Autonomie Longévité des Pays de la Loire, Conseil Régional des Pays de la Loire, Centre d’Expertise Partenariat Européen d’Innovation pour un Vieillissement Actif et en Bonne Santé, Nantes, France; 132 Department of Paediatrics and Child Health, University College Cork, Cork, Ireland; 133 Université Paris-Sud; Service de Pneumologie, Hôpital Bicêtre, Inserm UMR_S999, Le Kremlin Bicêtre, France; 134 Department of Medicine and Surgery, University of Salerno, Baronissi, Italy; 135 Servicio de Alergia e Immunologia, Clinica Santa Isabel, Buenos Aires, Argentina; 136 Hallym University College of Medicine, Hallym University Sacred Heart Hospital, Anyang, Gyeonggi-do South Korea; 137 Department of Clinical Immunology, Wrocław Medical University, Wrocław, Poland; 138 Ukrainian Medical Stomatological Academy, Poltava, Ukraine; 139 Pediatric Allergy and Asthma Unit, Hacettepe University School of Medicine, Ankara, Turkey; 140 First Department of Family Medicine, Medical University of Lodz, Łódź, Poland; 141 Institute of Social Medicine, Epidemiology and Health Economics, Charité-Universitätsmedizin Berlin, Berlin, Germany; 142 Institute for Clinical Epidemiology and Biometry, University of Wuerzburg, Würzburg, Germany; 143 National Research Center, Institute of Immunology, Federal Medicobiological Agency, Laboratory of Molecular Immunology, Moscow, Russian Federation; 144 GARD Chairman, Geneva, Switzerland; 145 Allergy & Asthma Center Westend, Berlin, Germany; 146 Department of Immunology and Allergy, Healthy Ageing Research Center, Medical University of Lodz, Łódź, Poland; 147 Department of Clinical Science and Education, Södersjukhuset, Karolinska Institutet, Stockholm, Sweden; 148 Sach’s Children and Youth Hospital, Södersjukhuset, Stockholm, Sweden; 149 Mattone Internazionale Program, Veneto Region, Italy; 150 Departments of Internal Medicine and Pediatrics (Divisions of Allergy and Immunology), University of Tennessee College of Medicine, Germantown, TN USA; 151 Scottish Centre for Respiratory Research, Cardiovascular & Diabetes Medicine, Medical Research Institute, Ninewells Hospital, University of Dundee, Dundee, UK; 152 Department of Paediatrics, Oslo University Hospital, Oslo, Norway; 153 Faculty of Medicine, Institute of Clinical Medicine, University of Oslo, Oslo, Norway; 154 Imunoalergologia, Centro Hospitalar Universitário de Coimbra and Faculty of Medicine, University of Coimbra, Coimbra, Portugal; 155 Department of Pulmonary Medicine, CHU Sart-Tilman, and GIGA I3 Research Group, Liege, Belgium; 156 DG for Health and Social Care, Scottish Government, Edinburgh, UK; 157 Department of Pulmonary Medicine, Rashid Hospital, Dubai, UAE; 158 Coimbra Institute for Clinical and Biomedical Research (iCBR), Faculty of Medicine, University of Coimbra, Coimbra, Portugal; 159 Department of Medicine (RCSI), Bon Secours Hospital, Glasnevin, Dublin, Ireland; 160 Kronikgune, International Centre of Excellence in Chronicity Research Barakaldo, Barakaldo, Bizkaia Spain; 161 Division of Clinical Immunology and Allergy, Laboratory of Behavioral Immunology Research, The University of Mississippi Medical Center, Jackson, MS USA; 162 Tobacco Control Research Centre, Iranian Anti Tobacco Association, Tehran, Iran; 163 Argentine Association of Allergy and Clinical Immunology, Buenos Aires, Argentina; 164 Department of Pediatric Pneumology and Immunology, AG Molecular Allergology and Immunomodulation, Charité Medical University, Berlin, Germany; 165 Institute of Environmental Medicine, Karolinska Institutet, Stockholm, Sweden; 166 PNDR, Portuguese National Programme for Respiratory Diseases, Faculdade de Medicina de Lisboa, Lisbon, Portugal; 167 Allergy and Asthma Medical Group and Research Center, San Diego, CA USA; 168 Department of Physiology, CHRU, University Montpellier, Vice President for Research, PhyMedExp, INSERM U1046, CNRS, UMR 9214, Montpellier, France; 169 Croatian Pulmonary Society, Zagreb, Croatia; 170 National Institute of Pneumology M Nasta, Bucharest, Romania; 171 Clinic for Pulmonary Diseases, Clinical Center of Serbia, Faculty of Medicine, University of Belgrade, Serbian Association for Asthma and COPD, Belgrade, Serbia; 172 Regione Piemonte, Turin, Italy; 173 Mexico City, Mexico; 174 National Center for Research in Chronic Respiratory Diseases, Tishreen University School of Medicine, Latakia, Syria; 175 Syrian Private University, Damascus, Syria; 176 Faculty of Medicine and Surgery, University of Medicine, La Valette, Malta; 177 Department of Medical Sciences, Allergy and Clinical Immunology Unit, University of Torino & Mauriziano Hospital, Turin, Italy; 178 Allergy Center, CUF Descobertas Hospital, Lisbon, Portugal; 179 Institute of Medical Statistics, and Computational Biology, Medical Faculty, University of Cologne, Cologne, Germany; 180 CRI-Clinical Research International-Ltd, Hamburg, Germany; 181 Danish Commitee for Health Education, Copenhagen East, Denmark; 182 Food Allergy Referral Centre Veneto Region, Department of Women and Child Health, Padua General University Hospital, Padua, Italy; 183 MedScript Ltd, Paraparomu, New Zealand; 184 OPC, Cambridge, UK; 185 Johns Hopkins School of Medicine, Baltimore, MD USA; 186 Consortium of Pharmacies and Services COSAFER, Salerno, Italy; 187 Scientific Centre of Children’s Health under the Russian Academy of Medical Sciences, Moscow, Russia; 188 Center of Allergy, Immunology and Respiratory Diseases, Santa Fe, Argentina; 189 Center for Allergy and Immunology, Santa Fe, Argentina; 190 Hospital of the Hospitaller Brothers in Buda, Budapest, Hungary; 191 Die Hautambulanz and Rothhaar Study Center, Berlin, Germany; 192 Department of Pharmacy, University of Naples Federico II, Naples, Italy; 193 ENT Department, University Hospital of Kinshasa, Kinshasa, Congo; 194 Department of Allergy, Immunology and Respiratory Medicine, Alfred Hospital and Central Clinical School, Monash University, Melbourne, VIC Australia; 195 Dept of Otolaryngology, Nippon Medical School, Tokyo, Japan; 196 Centre Hospitalier Universitaire Pédiatrique Charles de Gaulle, Ouagadougou, Burkina Faso; 197 Dept of Comparative Medicine, Messerli Research Institute of the University of Veterinary Medicine and Medical University, Vienna, Austria; 198 EFA European Federation of Allergy and Airways Diseases Patients’ Associations, Brussels, Belgium; 199 Department of Immunology and Allergology, Faculty of Medicine and Faculty Hospital in Pilsen, Charles University in Prague, Pilsen, Czech Republic; 200 Department of Allergy and Clinical Immunology, Ajou University School of Medicine, Suwon, South Korea; 201 Laboratoire HP2, Université Grenoble Alpes, Grenoble, France; 202 INSERM, U1042 and CHU de Grenoble, Grenoble, France; 203 Allergy Unit, CUF-Porto Hospital and Institute, Porto, Portugal; 204 Center for Research in Health Technologies and Information Systems, CINTESIS, Universidade do Porto, Porto, Portugal; 205 Maladies Infectieuses et immunitaires, CHUL, Quebec City, QC Canada; 206 Department of Otorhinolaryngology, Head and Neck Surgery, Section of Rhinology and Allergy, University Hospital Marburg, Philipps-Universität Marburg, Marburg, Germany; 207 Farmacias Holon, Lisbon, Portugal; 208 Centre for Empowering Patients and Communities, Faulkland, Somerset, UK; 209 Children’s Hospital Srebrnjak, Zagreb, Croatia; 210 School of Medicine, University J.J. Strossmayer, Osijek, Croatia; 211 University Hospital ‘Sv Ivan Rilski’, Sofia, Bulgaria; 212 Academic Centre of Primary Care, University of Aberdeen, Aberdeen, Scotland UK; 213 Research in Real-Life, Cambridge, UK; 214 Department of Otorhinolaryngology, University of Crete School of Medicine, Heraklion, Greece; 215 Department of Nephrology and Endocrinology, Karolinska University Hospital, Stockholm, Sweden; 216 Allergy Unit, Presidio Columbus, Catholic University of Sacred Heart, Rome, Italy; 217 IRCCS Oasi Maria SS, Troina, Italy; 218 Hospital de Clinicas, University of Parana, Paraná, Brazil; 219 Division of Allergy Asthma and Clinical Immunology, Emek Medical Center, Afula, Israel; 220 Allergy and Respiratory Research Group, The University of Edinburgh, Edinburgh, UK; 221 Association of Finnish Pharmacists, Helsinki, Finland; 222 Allergy and Clinical Immunology Department, Centro Médico-Docente la, Trinidad and Clínica El Avila, Caracas, Venezuela; 223 Sociedad Paraguaya de Alergia Asma e Inmunologia, Asunción, Paraguay; 224 Division of Allergy, Clinical Immunology and Rheumatology, Department of Pediatrics, Federal University of São Paulo, São Paulo, Brazil; 225 European Health Futures Forum (EHFF), Dromahair, UK; 226 Kyrgyzstan National Centre of Cardiology and Internal Medicine, Euro-Asian Respiratory Society, Bishkek, Kyrgyzstan; 227 Department of Respiratory Medicine, University Hospital Olomouc, Olomouc, Czech Republic; 228 Pulmonary Division, Heart Institute (InCor), Hospital da Clinicas da Faculdade de Medicina da Universidade de Sao Paulo, Sao Paulo, Brazil; 229 Department of Respiratory Medicine, Hvidovre Hospital & University of Copenhagen, Copenhagen, Denmark; 230 RNSA (Réseau National de Surveillance Aérobiologique), Brussieu, France; 231 Sidkkids Hospitala and Institute of Health Policy, Management and Evaluation, Toronto, Canada; 232 Department of ENT, Medical University of Graz, Graz, Austria; 233 Pneumology and Allergy Department CIBERES and Clinical & Experimental Respiratory Immunoallergy, IDIBAPS, University of Barcelona, Barcelona, Spain; 234 Division of Immunopathology, Department of Pathophysiology and Allergy Research, Center for Pathophysiology, Infectiology and Immunology, Medical University of Vienna, Vienna, Austria; 235 NRC Institute of Immunology FMBA of Russia, Moscow, Russia; 236 Laboratory of Immunopathology, Department of Clinical Immunology and Allergy, Sechenov First Moscow State Medical University, Moscow, Russia; 237 Montevideo, Uruguay; 238 Department of Public Health & Primary Care, Leiden University Medical Center (LUMC), Leiden, The Netherlands; 239 Department of Obstetrics and Gynaecology, Erasmus MC, University Medical Center, Rotterdam, The Netherlands; 240 Department of Chest Medicine, Centre Hospitalier Universitaire UCL Namur, Université Catholique de Louvain, Yvoir, Belgium; 241 Pulmonary Unit, Department of Medical Specialties, Arcispedale SMaria Nuova/IRCCS, AUSL di Reggio Emilia, Reggio Emilia, Italy; 242 Pulmonary Environmental Epidemiology Unit, CNR Institute of Clinical Physiology, Pisa, Italy; 243 CNR Institute of Biomedicine and Molecular Immunology “A Monroy”, Palermo, Italy; 244 Nova Southeastern University, Fort Lauderdale, FL USA; 245 Dept of Otorhinolaryngology, HNO-Klinik, Universitätsklinikum Düsseldorf, Düsseldorf, Germany; 246 Department of Otolaryngology, Yong Loo Lin School of Medicine, National University of Singapore, Singapore, Singapore; 247 Department of Medicine, Clinical Immunology and Allergy, McMaster University, Hamilton, ON Canada; 248 Centre for Clinical Research Sörmland, Uppsala University, Eskilstuna, Sweden; 249 Eshelman School of Pharmacy, University of North Carolina, Chapel Hill, NC USA; 250 Cyprus International Institute for Environmental & Public Health in Association with Harvard School of Public Health, Cyprus University of Technology, Limassol, Cyprus; 251 Department of Pediatrics, Hospital “Archbishop Makarios III”, Nicosia, Cyprus; 252 Department of Pulmonary Diseases, Celal Bayar University, Faculty of Medicine, Manisa, Turkey; 253 The Allergy and Asthma Institute, Islamabad, Pakistan; 254 Department of Paediatrics and Child Health, Red Cross Children’s Hospital, Cape Town, South Africa; 255 MRC Unit on Child & Adolescent Health, University of Cape Town, Cape Town, South Africa; 256 Bull DSAS, Echirolles, France; 257 Universidad Católica de Córdoba, Córdoba, Argentina; 258 Department of Otolaryngology Head and Neck Surgery, Beijing TongRen Hospital and Beijing Institute of Otolaryngology, Beijing, China; 259 State Key Laboratory of Respiratory Diseases, Guangzhou Institute of Respiratory Disease, The First Affiliated Hospital of Guangzhou Medical University, Guangzhou, China; 260 University Clinic of Respiratory and Allergic Diseases, Golnik, Slovenia; 261 National Hospital Organization, Tokyo National Hospital, Tokyo, Japan

**Keywords:** Health care transformation, Care pathways, Rhinitis, ARIA, MASK, POLLAR

## Abstract

**Background:**

In all societies, the burden and cost of allergic and chronic respiratory diseases are increasing rapidly. Most economies are struggling to deliver modern health care effectively. There is a need to support the transformation of the health care system into integrated care with organizational health literacy.

**Main body:**

As an example for chronic disease care, MASK (Mobile Airways Sentinel NetworK), a new project of the ARIA (Allergic Rhinitis and its Impact on Asthma) initiative, and POLLAR (Impact of Air POLLution on Asthma and Rhinitis, EIT Health), in collaboration with professional and patient organizations in the field of allergy and airway diseases, are proposing real-life ICPs centred around the patient with rhinitis, and using mHealth to monitor environmental exposure. Three aspects of care pathways are being developed: (i) Patient participation, health literacy and self-care through technology-assisted “patient activation”, (ii) Implementation of care pathways by pharmacists and (iii) Next-generation guidelines assessing the recommendations of GRADE guidelines in rhinitis and asthma using real-world evidence (RWE) obtained through mobile technology. The EU and global political agendas are of great importance in supporting the digital transformation of health and care, and MASK has been recognized by DG Santé as a Good Practice in the field of digitally-enabled, integrated, person-centred care.

**Conclusion:**

In 20 years, ARIA has considerably evolved from the first multimorbidity guideline in respiratory diseases to the digital transformation of health and care with a strong political involvement.

**Electronic supplementary material:**

The online version of this article (10.1186/s13601-019-0279-2) contains supplementary material, which is available to authorized users.

## Background

In all societies, the burden and cost of non-communicable diseases (NCDs) are increasing rapidly as advances in sanitation, public health measures and clinical care result in changes in demography [1]. Most, if not all, economies are struggling to deliver modern health care effectively [2]. Budgets will continue to be challenged with the move towards universal health coverage as demand increases and newer, more expensive technologies become available [3–5]. Traditional programmes, heavily reliant on specialist and supporting services, are becoming unaffordable. Innovative solutions are required to alleviate system wide pressures [6, 7]. There is a need to support authorities in the transformation of the health care system into integrated care with organizational health literacy [8].

Integrated care pathways (ICPs) are structured multi-disciplinary care plans detailing the key steps of patient care [9]. They promote the translation of guideline recommendations into local protocols and their application to clinical practice. They may be of particular interest in patients with multimorbidities since guidelines rarely consider them appropriately [10, 11]. An ICP forms all or part of the clinical record, documents the care given, and facilitates the evaluation of outcomes for continuous quality improvement [12]. ICPs should be carried out by a multidisciplinary team including physicians, pharmacists [13, 14] and allied health care professionals [15]. ICPs should integrate recommendations from clinical practice guidelines, but they usually (i) enhance recommendations by combining interventions, integrating quality assurance and (ii) describe co-ordination of care. Self-care and shared decision making are at the forefront of ICPs with the aim of empowering patients and their (professional and lay) care givers.

Rhinitis and asthma multimorbidity can be used as a model for chronic diseases since there is a broad agreement on the ‘gold standard’ of care [16–18]. In allergic rhinitis (AR) and asthma, adherence to treatment is a major unresolved problem [19, 20]. The vast majority of physicians prescribe regular treatment but patients (and physicians when they are allergic [21]) do not adhere to the advice. Instead of they self-treat based on personal experience as suggested by real-world data [19, 22]. There is thus a major disconnect between physicians and patients, either because of the clinical approach utilised or due to a lack of patient health literacy, with insufficient shared decision making (SDM). On-demand (prn) approaches are now proposed in both diseases [23–25] and represent a major change from previous recommendations. This new approach should be integrated in ICPs, but it needs to be applied to self-management and based on solid evidence.

ICPs have been proposed with a focus on new technologies that, through personally-held data on tablet devices and recording of ‘symptom load’, should enhance self-management and adherence to guidelines and ICPs. The science of supporting self-care and ICPs through mobile devices (mHealth) is in its infancy, but preliminary results are encouraging [26–28]. In the context of asthma, a systematic review showed that mobile apps were generally as effective as traditional models of supported self-management, but that they may be preferred in some clinical and demographic contexts as being convenient as well as efficient for the patient and the professional [29]. Standardisation and the establishment of the Privacy Code of Conduct for mHealth apps [25] will be important in ensuring patients on the safeguard of their data and in helping them choose reliable technological tools, which will be essential for ICP implementation.

As an example for chronic disease care, a new development of the ARIA initiative (ARIA phase 4) [30], along with POLLAR (Impact of Air POLLution on Asthma and Rhinitis), in collaboration with professional and patient organizations in the field of allergy and airway diseases, are proposing real-life ICPs centred around the patient with rhinitis, and using mHealth to monitor environmental exposure.

**Fig. 1:**
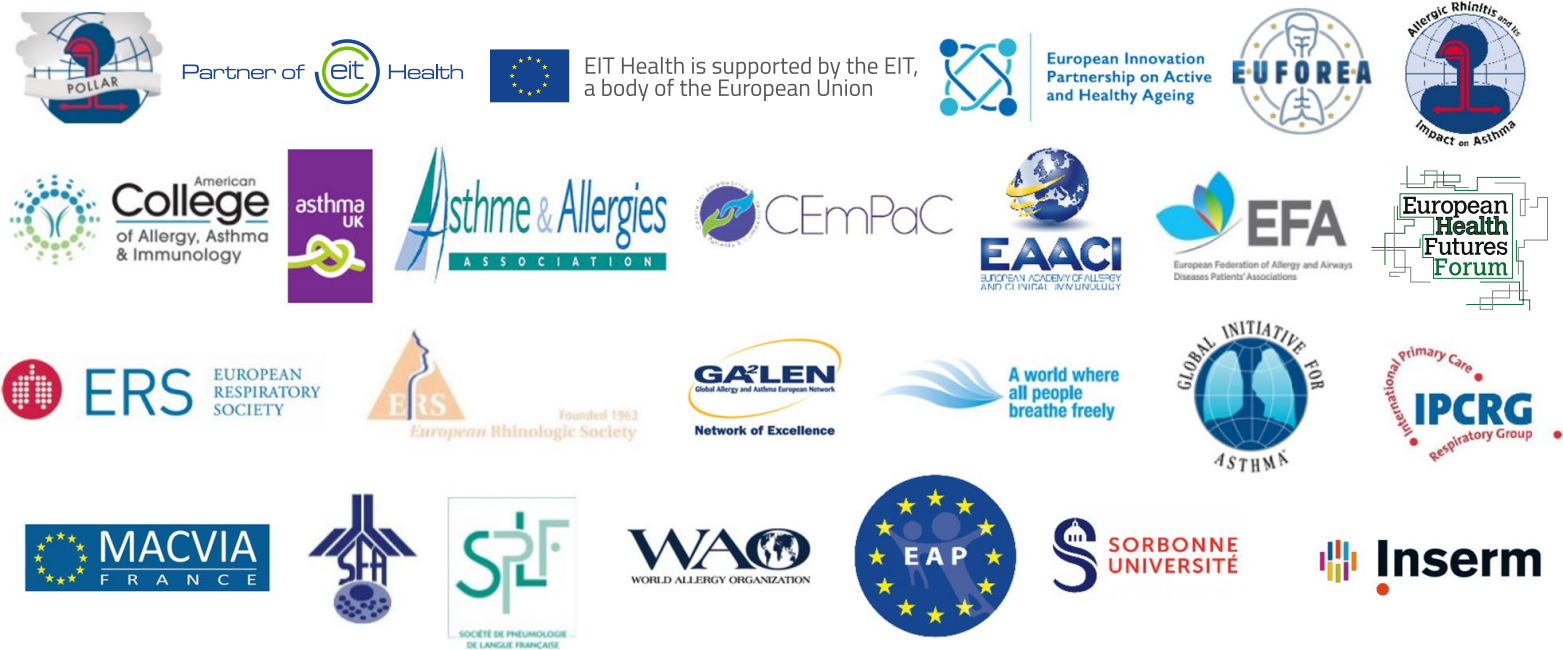
Organizations supporting the meeting

The current document was finalized and reviewed during a meeting involving ARIA, POLLAR (Impact of Air POLLution on Asthma and Rhinitis (EIT Health)), the European Innovation Partnership on Active and Healthy Ageing and the Global Alliance against Chronic Respiratory Diseases (GARD, WHO Alliance). Major allergy societies and patient’s organizations participated in this meeting (Paris, December 3, 2018). The event was carried out with the support of many organizations (Fig. 1).

## The gaps in allergic rhinitis and asthma

AR is the most common chronic disease worldwide. Treatment guidelines have improved the knowledge on rhinitis and have had a significant impact on AR management. However, many patients still fail to achieve sufficient symptom control [31] and the costs for society are enormous, in particular due to a major impact on school and work productivity [32] and on allergic or non-allergic multimorbidities [33, 34]. Allergic Rhinitis and its Impact on Asthma (ARIA) has promoted the use of its recommendations [16, 35, 36] to be integrated in ICPs using mobile technology in AR and asthma multimorbidity across the life cycle [37].

**Fig. 2:**
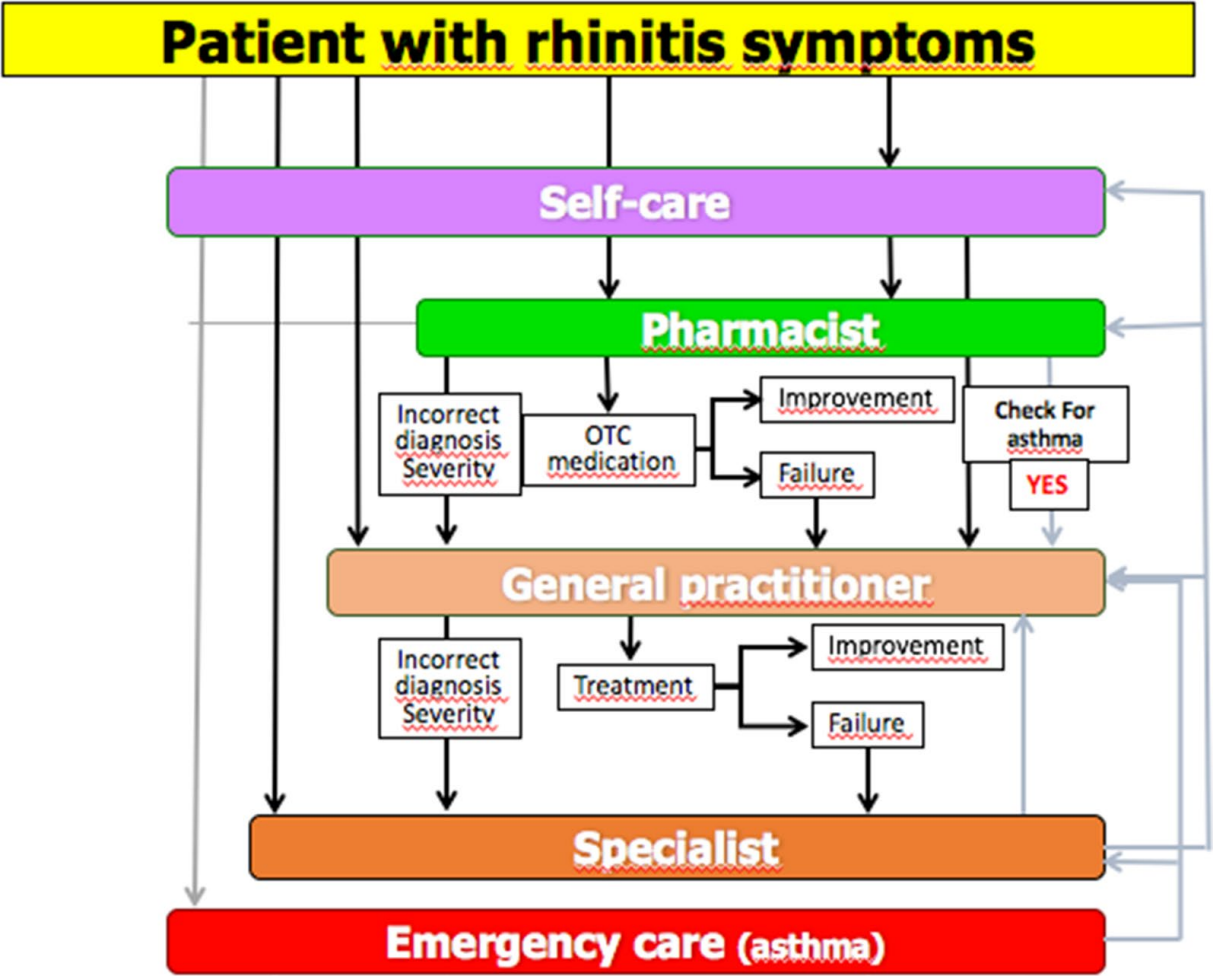
ICPs for rhinitis and asthma multimorbidity (adapted from [102])

The clinical problem is that a large number of AR patients do not consult physicians because they think their symptoms are ‘normal’ and/or trivial, even though AR negatively impacts social life, school and work productivity [36]. Many AR patients rely on over-the-counter (OTC) drugs and do not see the need to consult with physicians [38–41]. The vast majority of patients who visit general practitioners (GPs) or specialists have moderate-to-severe rhinitis [42–46]. ICPs should take this reality into account and consider a multi-disciplinary approach as proposed by AIRWAYS ICPs (Fig. 2).

## Supported self-management

People with AR and asthma are, by default, making day-to-day decisions about the management of their condition (avoiding triggers, using various treatments and seeking professional advice). Reflecting this broad concept, self-management is defined as “the tasks that individuals must undertake to live well with one or more chronic conditions. These tasks include having the confidence to deal with the medical management, role management and emotional management of their conditions” [47].

The term self-care includes generic “healthy lifestyle behaviours required for human development and functioning” [48]. However, self-care and self-management overlap as, for example, smoking cessation is a generic self-care behaviour and a component of self-management for people with respiratory conditions.

Self-management support is the assistance that professionals (pharmacy, primary care, specialist), patient’s organizations and other sources of information, as well as informal caregivers, give patients in order to make decisions about their condition and to manage disease and health-related tasks [49]. A taxonomy of 14 components of self-management support [50] offers a pick-list of activities that may be considered when planning self-management. These could be practical activities (e.g. teaching inhaler technique, discussing an action plan, helping to quit smoking) and imply SDM [50]. Mobile technology has the potential to contribute to many aspects of the supported self-management of chronic diseases [51].

Supported self-management is a ‘key principle’ for ICPs in long-term conditions [52, 53]. This not only reflects the paradigm shift towards SDM, but also includes pragmatic, economic imperatives, as healthcare systems respond to the increasing NCD burden. The economic impact of effective supported self-management goes beyond healthcare savings. For example, major economic return can be in the workplace where absenteeism and, more importantly, presenteeism are reduced [32] leading to an increased productivity.

Patient activation, defined as the “knowledge, skills and confidence a person has in managing his/her own health and health care” [54], is a goal of many ICP models. “Activation” encompasses the patients’ beliefs about their ability to self-manage (self-efficacy) and the likelihood that they will put these beliefs into action. Levels of activation range from the disengaged patients who let others manage their condition to the fully “activated” patients who embrace SDM and manage their health in partnership with their healthcare advisors, understanding the escalation of treatment options and when to seek pharmacy or medical advice. Higher levels of activation have been associated with better process and health outcomes in adults [55] and there is some evidence that appropriately-targeted self-management support may be more beneficial to disadvantaged groups than to higher literacy/socioeconomic status patients.

Although ARIA appears to meet the patient’s needs, real-life data obtained using the *Allergy Diary* (MASK-air^®^) app from around 10,000 people in 23 countries (Argentina, Austria, Australia, Belgium, Brazil, Canada, Czech Republic, Denmark, Finland, France, Germany, Greece, Italy, Lithuania, Mexico, Netherlands, Poland, Portugal, Spain, Sweden, Switzerland, UK. Brazil is a developing country) have shown that very few patients are being treated according to guidelines and that they often self-medicate [19]. Self-medication is the treatment of common health problems with medicines without medical supervision. It is important to ensure that well-written, short and accurate self-management information is available for people to pick up in pharmacies, or for download. In the case of AR, many patients have prescribed medications at home and, when symptoms occur, they use them. Self-care and SDM centred around the patient should be used more often [56]. ARIA has already followed a change management (CM) strategy embedding the AR-asthma multimorbidity in every day practice [30], but a new CM is now being considered to increase the benefits of self-care and SDM in ICPs using currently-available IT tools. In the case of AR and asthma multimorbidity, aeroallergen exposure and pollution impact disease control and medications. However, there is currently no ICP in airway diseases that takes such environmental parameters into account [57]. These initiatives should prepare and support individuals, teams and organizations in making organizational change centred around the patient.

mHealth, such as apps running on consumer smart devices, is becoming increasingly popular and has the potential to profoundly affect health care and health outcomes [58]. Several apps exist for AR and asthma [59–63]. A review of the Apps in the field of allergic diseases has recently been completed (Matricardi et al. in preparation). One of the reviews—MASK (Mobile Airways Sentinel NetworK), the Phase 3 ARIA initiative [37, 64]—is based on the freely-available MASK app (*the Allergy Diary*, Android and iOS platforms) for AR and asthma. Importantly, MASK is available in 17 languages and deployed in 23 countries [64]. Data from 26,000 users reporting over 200,000 days of treatment are available. It complies with the recent General Data Protection Regulation (EU) 2016/679 (GDPR) enforced by the EU, May 25, 2018 [65]. The GDPR aims primarily to give control to citizens and residents over their personal data and to simplify the regulatory environment by unifying the regulation within the EU [66, 67]. Importantly, MASK enables the assessment of treatment patterns in real life and provides detailed information on treatment, given that the *Allergy Diary* is able to distinguish between AR medications [19].

## On-line information

Most patients check on-line to help them decide what the problem is and how to address it. This is a crucial self-management area of support and we need to think about how it can be optimized. Because of the multiplicity of sources and the lack of reliability control, it should be recognized that such a task would require an enormous effort. Consequently, it has been abandoned by many other bodies/disease areas. One approach that may be of value in improving reliability would be to focus on sites that provide useful information and generate an accreditation process with international standing.

## Pharmacist care

Pharmacists are trusted health care professionals. Most patients with rhinitis are seen by pharmacists who are the initial point of contact of AR management in most countries. Depending on the country, few or most AR medications are available over-the-counter (OTC) [68–71] and are used by many patients. Therefore, as trusted health care professionals in the community, pharmacists are well placed to play a critical role identifying the symptoms of AR, recommending appropriate OTC treatment [38, 39, 41] and integrating health care teams through ICPs [13, 14]. The specific role of pharmacists in the management of AR within ICPs can been evidenced from several strategies that have been initiated [72] or completed and from studies confirming the important impact of pharmacist interventions on AR outcomes [40, 70, 73–80].

ARIA in the pharmacy 2004 [38] is being revised in order to propose ICPs involving a multi-disciplinary approach. This paper has been built on the evidence and provides tools intended to help pharmacists give optimal advice/interventions/strategies to patients with rhinitis. The ARIA-pharmacy ICP includes a diagnostic questionnaire specifically focusing attention on key symptoms and markers of the disease, a systematic Diagnosis Guide (including a differential diagnosis) and a simple flowchart proposing treatment for rhinitis and asthma multimorbidity. Key prompts for referral within the ICP are included. The use of technology is critical for enhancing the management of AR. The ARIA-pharmacy ICP should be adapted to local health care environments/situations as large regional or national differences in pharmacy-based care exist.

## Next-generation guidelines

Practice guidelines contain evidence-based statements about treatment, tests, public health actions and policy decisions intended to assist recipients of care and their care providers in making informed decisions.

ARIA was one of the first chronic respiratory disease guidelines to adopt the GRADE (Grading of Recommendation, Assessment, Development and Evaluation) approach, an advanced evidence evaluation and development approach for guidelines [16, 81–83]. GRADE-based guidelines are available for AR from other organizations and their recommendations are similar [16–18]. However, a limitation of GRADE is that evidence often lacks applicability because the populations studied do not reflect most of the patients seen in primary care [84]. The GRADE recommendations are often based on RCT in which patients regularly use their treatment, whereas most AR or asthma patients are non-adherent. GRADE rarely includes recommendations based on implementation research.

The more recently completed work by the GRADE working group on its Evidence to Decision Frameworks requires that guideline developers regularly address implementation and monitoring strategies [85–89]. Searching for and synthesizing evidence of effective implementation strategies enabled the BTS/SIGN asthma guideline to make a recommendation on how supported self-management for asthma could be embedded into routine practice [90]. Strategies include proactively engaging and empowering patients, training and motivating professionals as well as providing an environment that promotes self-management and monitors implementation [91]. In AR, cluster-randomized controlled trials have confirmed the overall value of guidelines [92, 93]. However, there has been only one direct testing of individual guideline recommendations in real–life studies in an effort to achieve optimization [94].

Next-generation ARIA-GRADE guidelines should consider testing the recommendations based on the GRADE approach with real-world evidence (RWE) using data obtained by mHealth tools such as MASK in order to confirm the efficiency or to refine current GRADE-based recommendations. The first results of MASK confirm the feasibility of the project [19]. Adherence to treatment is very low as < 5% of users record symptoms and medications for a period of 2 weeks. This indicates that it is important to further test whether on-demand is equally or even more efficient than regular-continuous treatment and that guidelines should consider both regular and on-demand treatment [19, 95].

Guideline recommendations often address isolated questions or focus on a single disease or problem. They should be considered in the context of the many decisions that are made. ICPs try to address the multiple options and iterative changes in a patient’s status and problems. Guideline recommendations should support these iterative changes.

The key challenge for conventional treatment guidelines is that available evidence, both from randomized trials and non-randomized studies, does not usually address the complex pathways, but only affects isolated decision points within a pathway. For example, when an oral H_1_-antihistamine is not achieving symptom control, we propose to replace it by an intra-nasal corticosteroid. However, this is often not the way that studies are designed and not how patients use these medications. Assuming that properly developed pathways require evidence, our guidelines must start identifying the best available evidence to support decision points. When the evidence is indirect, which is frequently the case, connecting the relevant decision points and considering all of that evidence together results in low certainty on the overall structure and timing of an ICP.

The next-generation guidelines, if complemented by the intelligent use of tools such as MASK, which records patients’ symptoms and provides advice at given time points to follow ICPs, could exemplify unique new tools to both implement and evaluate recommendations in the context of pathways. Studies should be carried out in which patients are randomized to ICPs or to follow ARIA recommendations that are not presented as pathways. Such studies will provide both information on the use of the recommendations and on the usefulness of the pathways. Through implementation of recommendations, we will be able to increase our certainty in the evidence by evaluating the entire pathway and measuring outcomes in direct population-based studies that record what patients do as opposed to what clinicians prescribe (and patients do not do).

## Study proposals of ARIA phase 4 and POLLAR

ARIA Phase 4 is the change management strategy for AR and asthma [96]. POLLAR is an EIT-Health (European Institute for Innovation and technology) project which aims to better understand, prevent and manage the impact of air pollution and allergen exposure on airway diseases [57]. POLLAR will use the MASK App, which is a Good Practice [64]. One of the POLLAR work-packages is the development of ICPs integrating aerobiology and air pollution. This will be developed using a step-wise approach centred around the patient. The four-step project is a WHO Global Alliance against Chronic Respiratory Diseases (GARD) demonstration project.

### Step 1: First meeting (December 3, 2018, Paris): Development of next-generation ICPs with a focus on self-management, pharmacy care and next-generation guidelines

**Fig. 3:**
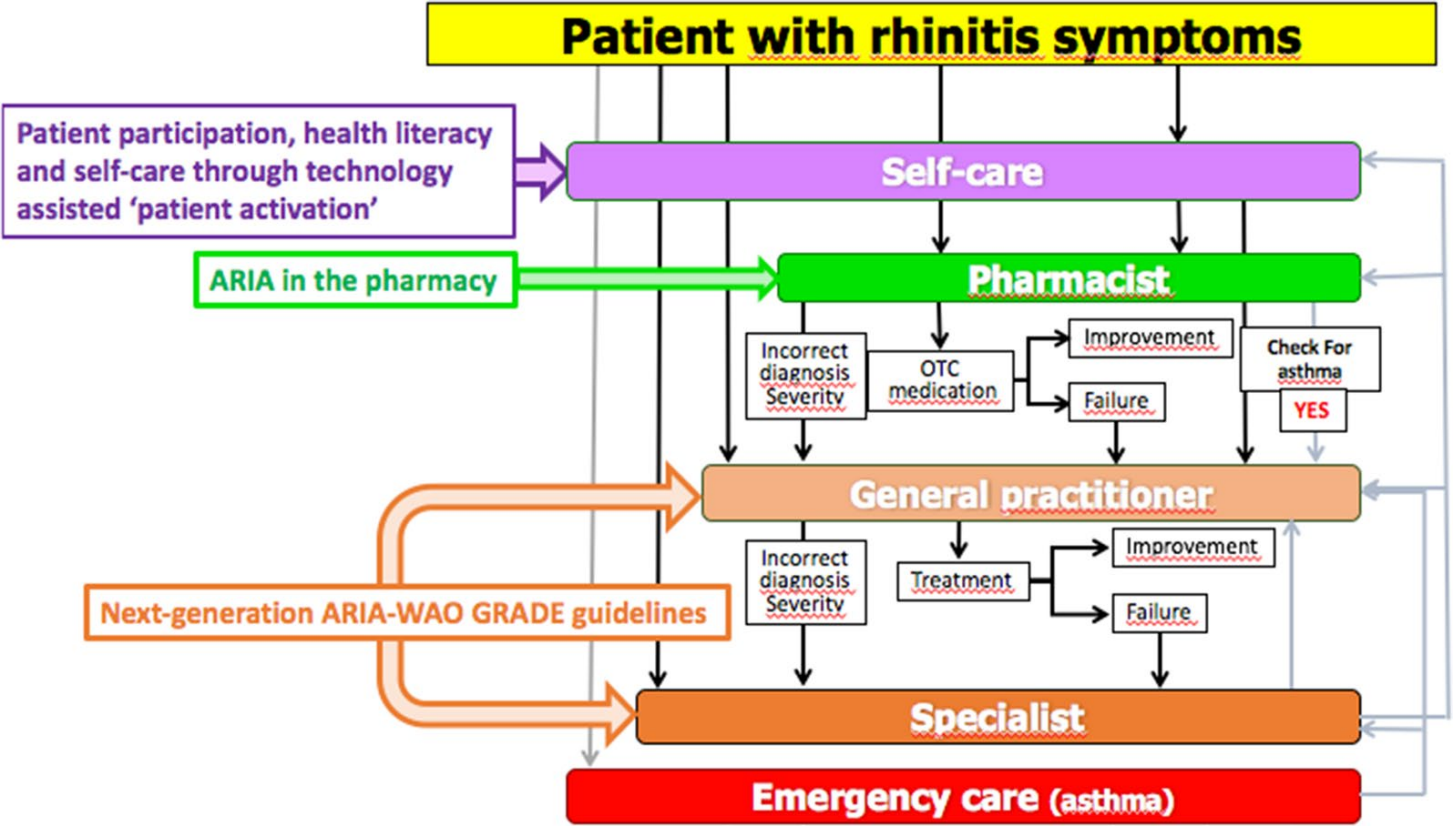
Next-generation ICPs for rhinitis and asthma multi-morbidity

The Paris meeting addressed a number of areas as delineated below (Fig. 3).

### Step 2: 2019–2021: Further development and implementation of next-generation ICPs

1. Develop a strategic and practical approach to improving patient autonomy and self-management programmes.
2. Deploy to other chronic respiratory diseases (asthma, COPD and rhinosinusitis [97]) and NCDs developing a multimorbidity App based on MASK expertise and experience.
3. Develop documents for specific age groups: pre-school and school children, older adults.
4. Establish a best practice across several regions in the EU linking the study to policy makers aiming to improve air quality and outcomes in their population.

### Step 3: Second meeting (December 2019): Embedding environmental data in next-generation ICPs

**Fig. 4:**
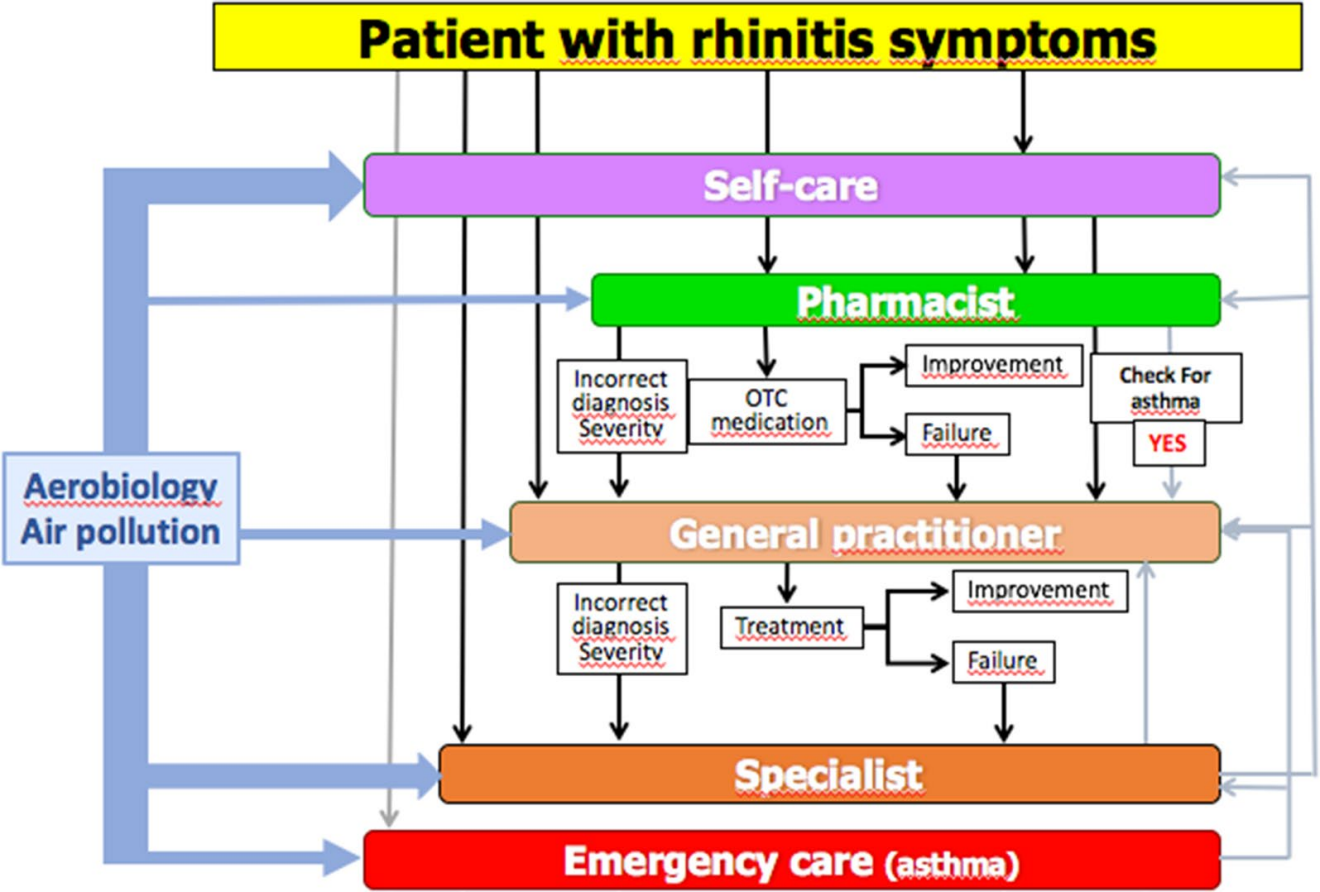
Embedding aerobiology and air pollution in ICPs

Using the results obtained by POLLAR for air pollution, a second meeting will be held to integrate aerobiology and air pollution data in mobile technology and to propose ICPs for the prevention of severe exacerbations and asthma during peaks of allergens and/or pollution. This meeting will also consider the deployment to other chronic diseases (Fig. 4) and the impact of biodiversity in chronic diseases [98].

## Embedding next-generation care pathways in the EU and global political agendas for allergic and chronic respiratory diseases

The Polish Presidency of the EU Council (2011) targeted CRDs in children to promote their early recognition, prevention and management to ultimately impact active and healthy ageing (AHA) [99]. The developmental determinants of CRDs in ageing were reinforced during the Cyprus Presidency of the EU Council “Healthy ageing across the lifecycle” (2012) [100] and an EU-NIH meeting held in Montpellier (2013) [101].

The objective of AIRWAYS-ICPs [102] was to launch a collaboration to develop multi-sectoral ICPs for CRDs in European countries and regions. AIRWAYS-ICPs was initiated in 2014 by the European Innovation Partnership on Active and Healthy Ageing (EIP on AHA, DG Santé and DG CONNECT) [103] as a GARD (Global Alliance against Chronic Respiratory Diseases) demonstration project [104]. In collaboration with GARD, the Directorate General of Health of Portugal, the EIP on AHA and the Région Occitanie (France), a high-level meeting was organized July 1, 2015 with all major European scientific societies and patient’s organizations in Lisbon to review the implementation results of AIRWAYS ICPs [105].

Euforea (European Forum for Research and Education in Allergy and Airway Diseases) [56] proposed an annual stepwise strategy at the EU or ministerial levels. A European Symposium on Precision Medicine in Allergy and Airways Diseases was held at the EU Parliament October 14, 2015 [106]. Another EU Parliament meeting was held in Brussels March 29, 2017 on the Prevention and Self-Management of CRDs using novel mobile health tools [37, 56, 97].

POLLAR (Impact of air POLLution on Asthma and Rhinitis, EIT Health) is focusing on the impact of allergens and air pollution on airway diseases and aims to propose novel ICPs integrating pollution, sleep and patients’ literacy and to assess the societal implications of the interaction [57].

Euforea organized an EU Summit in Vilnius, Lithuania (March 2018) in collaboration with the Ministers of Health of Lithuania, Moldova, Georgia and Ukraine. The aim was to discuss and start the implementation of the POLLAR concepts, and to deploy it to EU neighboring countries. The Vilnius Declaration on Chronic Respiratory Diseases proposed multisectoral ICPs embedding guided self-management, mHealth and air pollution in CRDs [107].

The joint meeting discussed in this report (December 3, 2018) proposed next-generation care pathways based on the Vilnius Declaration.

MASK has been selected by the European Commission’s Directorate-General for Health and Food Safety (DG SANTE) and the newly-established Commission Expert Group “Steering Group on Health Promotion, Disease Prevention and Management of Non-Communicable Diseases” as a Good Practice (GP) in the field of digitally-enabled, integrated, person-centred care.

On May 3, 2019, a Euforea-led meeting took place in the Parliament of Malta to review the results of the December 3 meeting and to propose practical strategies at the EU and global levels with GARD.

This new next-generation care pathway is completely aligned with the recommendations issued by the Thematic Network SHAFE—Smart Healthy Age-Friendly Environments (approved by the European Commission—DG SANTE and DG CONNECT)—on its Joint Statement delivered 12th November 2018. The Statement underlined the need to patient empowerment and active involvement in its healthcare process and also urged the use of lifestyle medicine that provides effective impact on the patient’s wellbeing.

## Conclusions

There is a need to support the digital transformation of health and care with integrated care. An innovative patient-centered approach is proposed by the ARIA expert group for rhinitis and asthma multimorbidity to be scaled up to chronic diseases.

## Additional file

**Additional file 1.** The MASK Study Group.

## Data Availability

Not applicable.

## Abbreviations

AIRWAYS ICPs: integrated care pathways for airway diseases
AR: allergic rhinitis
ARIA: Allergic Rhinitis and its Impact on Asthma
BTS/SIGN: British Thoracic Society/Scottish Intercollegiate Guidelines Network
CRD: chronic respiratory diseases
EIP on AHA: European Innovation Partnership on Active and Healthy Ageing
EIT-Health: European Institute for Innovation and technology-Health
Euforea: European Forum for Research and Education in Allergy and Airway Diseases
GARD: Global Alliance against Chronic Respiratory Diseases
GDPR: General Data Privacy Regulation
GLASS-ARIA: Global Allergy Simple Solution
GP: general practitioner
GRADE: Grading of Recommendation, Assessment, Development and Evaluation
ICP: integrated care pathways
MASK: Mobile Airways Sentinel Network
MHealth: mobile health
OTC: over-the-counter
PG: pocket guides
POLLAR: Impact of Air POLLution in Asthma and Rhinitis
RWE: Real World Evidence
SDM: shared decision making
WAO: World Allergy Organization
WHO: World Health Organization
