# Supplementary material for: Next-generation ARIA care pathways for rhinitis and asthma: a model for multimorbid chronic diseases
Source: Clin Transl Allergy. 2019 Sep 9;9:44. doi: 10.1186/s13601-019-0279-2 (PMC6734297; doi:10.1186/s13601-019-0279-2)
Supplement: Supplementary file 1 — Additional file 1. The MASK Study Group. [file 13601_2019_279_MOESM1_ESM.pdf]

MASK Study group

J Bousquet [1-3], PW Hellings [4], W Aberer [5], I Agache [6], CA Akdis [7], M Akdis [7], MR Aliberti [8], R Almeida[9], F Amat [10], R Angles [11], I Annesi-Maesano [12], IJ Ansotegui [13], JM Anto [14-17], S Arnavielle [18], E Asayag [19], A Asarnoj [20], H Arshad [21], F Avolio [22], E Bacci [23], C Bachert [24], I Baiardini [25], C Barbara [26], M Barbagallo [27], I Baroni [28], BA Barreto [29], X Basagana [14], ED Bateman [30], M Bedolla-Barajas [31], A Bedbrook [2], M Bewick [32], B Beghé [33], EH Bel [34], KC Bergmann [35], KS Bennoor [36], M Benson [37], L Bertorello [23], AZ Białoszewski [38], T Bieber [39], S Bialek [40], C Bindslev-Jensen [41] , L Bjermer [42], H Blain [43,44], F Blasi [45], A Blua [46], M Bochenska Marciniak [47], I Bogus-Buczynska [47], AL Boner [48], M Bonini [49], S Bonini [50], CS Bosnic-Anticevich [51], I Bosse [52], J Bouchard [53], LP Boulet [54], R Bourret [55], PJ Bousquet [12], F Braido [25], V Briedis [56], CE Brightling [57], J Brozek [58], C Bucca [59], R Buhl [60], R Buonaiuto [61], C Panaitescu [62], MT Burguete Cabañas [63] , E Burte [3], A Bush [64], F Caballero-Fonseca [65], D Caillaud [67], D Caimmi [68], MA Calderon [69], PAM Camargos [70], T Camuzat [71], G Canfora [72], GW Canonica [25], V Cardona [73], KH Carlsen [74], P Carreiro-Martins [75], AM Carriazo [76], W Carr [77], C Cartier [78], T Casale [79], G Castellano [80], L Cecchi [81], AM Cepeda [82], NH Chavannes [83], Y Chen [84], R Chiron [68], T Chivato [85], E Chkhartishvili [86], AG Chuchalin [87], KF Chung [88], MM Ciaravolo [89], A Ciceran [90], C Cingi [91], G Ciprandi [92], AC Carvalho Coehlo [93], L Colas [94], E Colgan [95], J Coll [96], D Conforti [97], J Correia de Sousa [98], RM Cortés-Grimaldo [99], F Corti [100], E Costa [101], MC Costa-Dominguez [102], AL Courbis [103], L Cox [104], M Crescenzo [105], AA Cruz [106], A Custovic [107], W Czarlewski [108], SE Dahlen [109], G D'Amato [381], C Dario [110], J da Silva [111], Y Dauvilliers [112], U Darsow [113], F De Blay [114], G De Carlo [115], T Dedeu [116], M de Fátima Emerson [117], G De Feo [118], G De Vries [119], B De Martino, [120] NP Motta Rubina [121], D Deleanu [122], P Demoly [12,68], JA Denburg [123], P Devillier [124], S Di Capua Ercolano [125], N Di Carluccio [66], A Didier [126], D Dokic [127], MG Dominguez-Silva [128], H Douagui [129], G Dray [103], R Dubakiene [130], SR Durham [131], G Du Toit [132], MS Dykewicz [133], Y El-Gamal [134], P Eklund [135], E Eller [41], R Emuzyte [136], J Farrell [95], A Farsi [81], J Ferreira de Mello Jr [137], J Ferrero [138], A Fink-Wagner [139], A Fiocchi [140], WJ Fokkens [141], JA Fonseca [142], JF Fontaine [143], S Forti [97], JM Fuentes-Perez [144], JL Gálvez-Romero [145], A Gamkrelidze [146], J Garcia-Aymerich [14], CY García-Cobas [147], MH Garcia-Cruz [148], B Gemicioğlu [149], S Genova [150], G Christoff [151], JE Gereda [152], R Gerth van Wijk [153], RM Gomez [154], J Gómez-Vera [155], S González Diaz [156], M Gotua [157], I Grisle [158], M Guidacci [159], NA Guldemond [160], Z Gutter [161], MA Guzmán [162] , T Haahtela [163], J Hajjam [164], L Hernández [165], JO'B Hourihane [166], YR Huerta-Villalobos [167], M Humbert [168], G Iaccarino [169], M Illario [170] , Z Ispayeva [380], JC Ivancevich [171], EJ Jares [172], E Jassem [173], SL Johnston [174], G Joos [175], KS Jung [176], J Just [10], M Jutel [177], I Kaidashev [178], O Kalayci [179], AF Kalyoncu [180], J Karjalainen [181], P Kardas [182], T Keil [183], PK Keith [184], M Khaitov [185], N Khaltaev [186], J Kleine-Tebbe [187], L Klimek [188], ML Kowalski [189], M Kuitunen [190], I Kull [191], P Kuna [47], M Kupczyk [47], V Kvedariene [192], E Krzych-Fałta [193], P Lacwik [47], D Larenas-Linnemann [194], D Laune [18], D Lauri [195], J Lavrut [196], LTT Le [197], M Lessa [198], G Levato [199], J Li [200], P Lieberman [201], A Lipiec [193], B Lipworth [202], KC Lodrup Carlsen [203], R Louis [204], O Lourenço [205], JA Luna-Pech [206] , A Magnan [94], B Mahboub [207], D Maier [208], A Mair [209], I Majer [210], J Malva [211], E Mandajieva [212], P Manning [213], E De Manuel Keenoy [214], GD Marshall [215], MR Masjedi [216], JF Maspero [217], E Mathieu-Dupas [18], JJ Matta Campos [218], AL Matos [219], M Maurer [220], S Mavale-Manuel [221], O Mayora [97], MA Medina-Avalos [222], E Melén [223], E Melo-Gomes [26], EO Meltzer [224], E Menditto [225], J Mercier [226], N Miculinic [227], F Mihaltan [228], B Milenkovic [229], G Moda [230], MD Mogica-Martinez [231], Y Mohammad [232], I Momas [233,234], S Montefort [235], R Monti [236], D Mora Bogado [237], M Morais-Almeida [238], FF Morato-Castro [239], R Mösges [240], A Mota-Pinto [241], P Moura Santo [242], J Mullol [243], L Münter [244], A Muraro [245], R Murray [246], R Naclerio [247], R Nadif [3], M Nalin [28], L Napoli [248], L Namazova-Baranova [249], H Neffen [250], V Niedeberger [251], K Nekam [252], A Neou [253], A Nieto [254], L Nogueira-Silva [255], M Nogues [2,256], E Novellino [257], TD Nyembue [258], RE O'Hehir [259], C Odzhakova [260], K Ohta [261], Y Okamoto [262], K Okubo [263], GL Onorato [2], M Ortega Cisneros [264], S Ouedraogo [265], I Pali-Schöll [266], S Palkonen [115], P Panzner [267], NG Papadopoulos [268], HS Park [269], A Papi [270], G Passalacqua [271], E Paulino [272], R Pawankar [273], S Pedersen [274], JL Pépin [275], AM Pereira [276], M Persico [277], O Pfaar [278,] r, J Phillips [280], R Picard [281], B Pigearias [282], I Pin [283], C Pitsios [284], D Plavec [285], W Pohl [286], TA Popov [287], F Portejoie [2], P Potter [288], AC Pozzi [289], D Price [290], EP Prokopakis [291], R Puy [259], B Pugin [292], RE Pulido Ross [293], M Przemecka [47], KF Rabe [294], F Raciborski [193], R Rajabian-Soderlund [295], S Reitsma [141], I Ribeirinho [296], J Rimmer [297], D Rivero-Yeverino [298], JA Rizzo [299], MC Rizzo [300], C Robalo-Cordeiro [301], F Rodenas [302], X Rodo [14], M Rodriguez Gonzalez, [303] , L Rodriguez-Mañas [304], C Rolland [305], S Rodrigues Valle [306] , M Roman Rodriguez [307], A Romano [308], E Rodriguez-Zagal [309], G Rolla [310], RE Roller-Wirnsberger [311], M Romano [28], J Rosado-Pinto [312], N. Rosario [313], M Rottem [314], D Ryan [315], H Sagara [316], J Salimäki [317], B Samolinski [193], M Sanchez-Borges [318], J Sastre-Dominguez [319], GK Scadding [320], HJ Schunemann [58], N Scichilone [321], P Schmid-Grendelmeier [322], FS Serpa [323], S Shamai [240], A Sheikh [324], M Sierra [96], FER Simons [325], V Siroux [326], JC Sisul [327], I Skrindo [378], D Solé [328], D Somekh [329], M Sondermann [330], T Sooronbaev [331], M Sova [332], M Sorensen, [333] M Sorlini [334], O Spranger [139], C Stellato [118], R Stelmach [335], R Stukas [336], J Sunyer [14-17], J Strozek [193], A Szylling [193], JN Tebyriçá [337], M Thibaudon [338], T To [339], A Todo-Bom [340], PV Tomazic [341], S Toppila-Salmi [163], U Trama [342], M Triggiani [118], C Suppli Ulrik [343], M Urrutia-Pereira [344], R Valenta [345], A Valero [346], A Valiulis [347], E Valovirta [348], M van Eerd [119], E van Ganse [349], M van Hage [350], O Vandenplas [351], MT Ventura [352], G Vezzani [353], T Vasankari [354], A Vatrella [118], MT Verissimo [211], F Viart [78], G Viegi [355], D Vicheva [356], T Vontetsianos [357], M Wagenmann [358], S Walker [359], D Wallace [360], DY Wang [361], S Waserman [362], T Werfel [363], M Westman [364], M Wickman [191], DM Williams [365], S

Williams [366], N Wilson [379], J Wright [367], P Wroczynski [40], P Yakovliev [368], BP Yawn [369], PK Yiallouros [370], A Yorgancioglu [371], OM Yusuf [372], HJ Zar [373], L Zhang [374], N Zhong [200], ME Zernotti [375], I Zhanat, [380], M Zidarn [376], T Zuberbier [35], C Zubrinich [259], A Zurkuhlen [377]

1. University Hospital, Montpellier, France.
2. MACVIA-France, Fondation partenariale FMC VIA-LR, Montpellier, France.
3. VIMA. INSERM U 1168, VIMA : Ageing and chronic diseases Epidemiological and public health approaches, Villejuif, Université Versailles St-Quentin-en-Yvelines, UMR-S 1168, Montigny le Bretonneux, France and Euforea, Brussels, Belgium.
4. Laboratory of Clinical Immunology, Department of Microbiology and Immunology, KU Leuven, Leuven, Belgium.
5. Department of Dermatology, Medical University of Graz, Graz, Austria.
6. Transylvania University Brasov, Brasov, Romania.
7. Swiss Institute of Allergy and Asthma Research (SIAF), University of Zurich, Davos, Switzerland.
8. Project Manager, Chairman of the Council of Municipality of Salerno, Italy.
9. Center for Health Technology and Services Research- CINTESIS, Faculdade de Medicina, Universidade do Porto; and Medida, Lda Porto, Portugal.
10. Allergology department, Centre de l'Asthme et des Allergies Hôpital d'Enfants Armand-Trousseau (APHP); Sorbonne Université, UPMC Univ Paris 06, UMR_S 1136, Institut Pierre Louis d'Epidémiologie et de Santé Publique, Equipe EPAR, Paris, France.
11. Innovación y nuevas tecnologías, Salud Sector sanitario de Barbastro, Barbastro, Spain.
12. Epidemiology of Allergic and Respiratory Diseases, Department Institute Pierre Louis of Epidemiology and Public Health, INSERM and Sorbonne Université, Medical School Saint Antoine, Paris, France
13. Department of Allergy and Immunology, Hospital Quirón Bizkaia, Erandio, Spain.
14. ISGlobAL, Centre for Research in Environmental Epidemiology (CREAL), Barcelona, Spain.
15. IMIM (Hospital del Mar Research Institute), Barcelona, Spain.
16. CIBER Epidemiología y Salud Pública (CIBERESP), Barcelona, Spain.
17. Universitat Pompeu Fabra (UPF), Barcelona, Spain.
18. KYomed INNOV, Montpellier, France.
19. Argentine Society of Allergy and Immunopathology, Buenos Aires, Argentina.
20. Clinical Immunology and Allergy Unit, Department of Medicine Solna, Karolinska Institutet, Stockholm, and Astrid Lindgren Children's Hospital, Department of Pediatric Pulmonology and Allergy, Karolinska University Hospital, Stockholm, Sweden.
21. David Hide Asthma and Allergy Research Centre, Isle of Wight, United Kingdom.
22. Regionie Puglia, Bari, Italy.
23. Regione Liguria, Genoa, Italy.
24. Upper Airways Research Laboratory, ENT Dept, Ghent University Hospital, Ghent, Belgium.
25. Allergy and Respiratory Diseases, Ospedale Policlinico San Martino, University of Genoa, Italy.
26. PNDR, Portuguese National Programme for Respiratory Diseases, Faculdade de Medicina de Lisboa, Lisbon, Portugal.
27. Director of the Geriatric Unit, Department of Internal Medicine (DIBIMIS), University of Palermo, Italy.
28. Telbios SRL, Milan, Italy.
29. Universidade do Estado do Pará, Belem, Brazil.
30. Department of Medicine, University of Cape Town, Cape Town, South Africa.
31. Hospital Civil de Guadalajara Dr Juan I Menchaca, Guadalarara, Mexico.
32. iQ4U Consultants Ltd, London, UK.
33. Section of Respiratory Disease, Department of Oncology, Haematology and Respiratory Diseases, University of Modena and Reggio Emilia, Modena, Italy.
34. Department of Respiratory Medicine, Academic Medical Center (AMC), University of Amsterdam, The Netherlands.
35. Charité - Universitätsmedizin Berlin, corporate member of Freie Universität Berlin, Humboldt-Universität zu Berlin, and Berlin Institute of Health, Comprehensive Allergy Center, Department of Dermatology and Allergy, Global Allergy and Asthma European Network (GA^2LEN), Berlin, Germany.
36. Dept of Respiratory Medicine, National Institute of Diseases of the Chest and Hospital, Dhaka, Bangladesh.
37. Centre for Individualized Medicine, Department of Pediatrics, Faculty of Medicine, Linköping, Sweden.
38. Department of Prevention of Environmental Hazards and Allergology, Medical University of Warsaw, Poland.
39. BIEBER. Department of Dermatology and Allergy, Rheinische Friedrich-Wilhelms-University Bonn, Bonn, Germany
40. Dept of Biochemistry and Clinical Chemistry, University of Pharmacy with the Division of Laboratory Medicine, Warsaw Medical University, Warsaw, Poland.

41. Department of Dermatology and Allergy Centre, Odense University Hospital, Odense Research Center for Anaphylaxis (ORCA), Odense, Denmark.
42. Department of Respiratory Medicine and Allergology, University Hospital, Lund, Sweden.
43. Department of Geriatrics, Montpellier University Hospital, Montpellier, France.
44. EA 2991, Euromov, University Montpellier, France.
45. Department of Pathophysiology and Transplantation, University of Milan, IRCCS Fondazione Ca'Granda Ospedale Maggiore Policlinico, Milan, Italy.
46. Argentine Association of Respiratory Medicine, Buenos Aires, Argentina.
47. Division of Internal Medicine, Asthma and Allergy, Barlicki University Hospital, Medical University of Lodz, Poland.
48. Pediatric Department, University of Verona Hospital, Verona, Italy.
49. UOC Pneumologia, Istituto di Medicina Interna, F. Policlinico Gemelli IRCCS, Università Cattolica del Sacro Cuore, Rome, Italy, and National Heart and Lung Institute, Royal Brompton Hospital & Imperial College London, UK.
50. Second University of Naples and Institute of Translational Medicine, Italian National Research Council.
51. Woolcock Institute of Medical Research, University of Sydney and Woolcock Emphysema Centre and Local Health District, Glebe, NSW, Australia.
52. Allergist, La Rochelle, France.
53. Associate professor of clinical medecine, Laval's University, Quebec city, Head of medecine department, Hôpital de la Malbaie, Quebec , Canada.
54. Quebec Heart and Lung Institute, Laval University, Québec City, Quebec, Canada.
55. Centre Hospitalier Valenciennes, France.
56. Head of Department of Clinical Pharmacy of Lithuanian University of Health Sciences, Kaunas, Lithuania.
57. Institute of Lung Health, Respiratory Biomedical Unit, University Hospitals of Leicester NHS Trust, Leicestershire, UK; Department of Infection, Immunity and Inflammation, University of Leicester, Leicester, UK.
58. Department of Health Research Methods, Evidence and Impact, Division of Immunology and Allergy, Department of Medicine, McMaster University, Hamilton, ON, Canada.
59. Chief of the University Pneumology Unit- AOU Molinette, Hospital City of Health and Science of Torino, Italy.
60. Universitätsmedizin der Johannes Gutenberg-Universität Mainz, Mainz, Germany.
61. Pharmacist, Municipality Pharmacy, Sarno, Italy.
62. University of Medicine and Pharmacy Victor Babes, Timisoara, Romania.
63. Instituto de Pediatria, Hospital Zambrano Hellion Tec de Monterrey, Monterrey, Mexico.
64. Imperial College and Royal Brompton Hospital, London, UK.
65. Centro Medico Docente La Trinidad, CaRacas, Venezuela.
66. Regional Director Assofarm Campania and Vice President of the Board of Directors of Cofaser, Salerno, Italy
67. Service de pneumologie, CHU et université d'Auvergne, Clermont-Ferrand, France.
68. Department of Respiratory Diseases, Montpellier University Hospital, France.
69. Imperial College London - National Heart and Lung Institute, Royal Brompton Hospital NHS, London, UK.
70. Federal University of Minas Gerais, Medical School, Department of Pediatrics, Belo Horizonte, Brazil
71. Assitant Director General, Montpellier, Région Occitanie, France.
72. Mayor of Sarno and President of Salerno Province, Director, Anesthesiology Service, Sarno "Martiri del Villa Malta" Hospital, Italy.
73. Allergy Section, Department of Internal Medicine, Hospital Vall d'Hebron & ARADyAL Spanish Research Network, Barcelona, Spain.
74. Department of Paediatrics, Oslo University Hospital and University of Oslo, Oslo, Norway.
75. CEDOC, Integrated Pathophysiological Mechanisms Research Group, Nova Medical School, Campo dos Martires da Patria, Lisbon, and Serviço de Imunoalergologia, Centro Hospitalar de Lisboa Central, EPE, Lisbon, Portugal.
76. Regional Ministry of Health of Andalusia, Seville, Spain.
77. Allergy and Asthma Associates of Southern California, Mission Viejo, CA, USA.
78. ASA - Advanced Solutions Accelerator, Clapiers, France.
79. Division of Allergy/Immunology, University of South Florida, Tampa, Fla, USA.
80. Celentano pharmacy, Massa Lubrense, Italy.
81. SOS Allergology and Clinical Immunology, USL Toscana Centro, Prato, Italy.
82. Allergy and Immunology Laboratory, Metropolitan University Hospital, Branquilla, Columbia.
83. Department of Public Health and Primary Care, Leiden University Medical Center, Leiden, The Netherlands
84. Capital Institute of Pediatrics, Chaoyang district, Beijing, China.
85. School of Medicine, University CEU San Pablo, Madrid, Spain.
86. David Tvildiani Medical University - AIETI Highest Medical School, David Tatishvili Medical Center Tbilisi, Georgia.
87. Pulmonolory Research Institute FMBA, Moscow, Russia and GARD Executive Committee, Moscow, Russia.

88. National Heart & Lung Institute, Imperial College, London, UK.
89. Specialist social worker, Sorrento, Italy.
90. Argentine Federation of Otorhinolaryngology Societies, Buenos Aires, Argentina.
91. Eskisehir Osmangazi University, Medical Faculty, ENT Department, Eskisehir,Turkey.
92. Medicine Department, IRCCS-Azienda Ospedaliera Universitaria San Martino, Genoa, Italy.
93. Universidade Federal da Bahia, Escola de Enfermagem, Brazil.

94. Plateforme Transversale d'Allergologie, Institut du Thorax, CHU de Nantes, Nantes, France.
95. LANUA International Healthcare Consultancy, Northern Ireland, UK.
96. Innovación y nuevas tecnologías, Salud Sector sanitario de Barbastro, Barbastro, Spain.
97. Innovation and Research Office, Department of Health and Social Solidarity, Autonomous Province of Trento, Italy.
98. Life and Health Sciences Research Institute (ICVS), School of Medicine, University of Minho, Braga, Portugal; ICVS/3B's, PT Government Associate Laboratory, Braga/Guimarães, Portugal.
99. Servicio de Allergologia, Hospital Angeles del Carmen, Guadalajara, Mexico.

100. FIMMG (Federazione Italiana Medici di Medicina Generale), Milan, Italy.
101. UCIBIO, REQUINTE, Faculty of Pharmacy and Competence Center on Active and Healthy Ageing of University of Porto (Porto4Ageing), Porto, Portugal.
102. Allergologo, Mexico City, Mexico.

103. IMT Mines Alès, Unversité Montpellier, Alès, France.
104. Department of Medicine, Nova Southeastern University, Davie, University of Miami Dept of Medicine, Miami, Florida, USA.
105. Regional Director Assofarm Campania and Vice President of the Board of Directors of Cofaser, Salerno, Italy.

106. ProAR – Nucleo de Excelencia em Asma, Federal University of Bahia, Brasil and WHO GARD Planning Group, Brazil.
107. Centre for Respiratory Medicine and Allergy, Institute of Inflammation and Repair, University of Manchester and University Hospital of South Manchester, Manchester, UK.
108. Medical Consulting Czarlewski, Levallois, France.
109. The Centre for Allergy Research, The Institute of Environmental Medicine, Karolinska Institutet, Stockholm, Sweden.
110. Azienda Provinciale per i Servizi Sanitari di Trento (APSS-Trento), Italy.
111. Department of Internal Medicine and Allergy Clinic of Professor Polydoro Ernani de São Thiago University Hospital, Federal University of Santa Catarina (UFSC), Florianópolis, Santa Catarina, Brazil.

112. Sleep Unit, Department of Neurology, Hôpital Gui-de-Chauliac Montpellier, Inserm U1061, France.
113. Department of Dermatology and Allergy, Technische Universität München, Munich, Germany; ZAUM-Center for Allergy and Environment, Helmholtz Center Munich, Technische Universität München, Munich, Germany.
114. Allergy Division, Chest Disease Department, University Hospital of Strasbourg, Strasbourg, France.
115. EFA European Federation of Allergy and Airways Diseases Patients' Associations, Brussels, Belgium
116. AQuAS, Barcelna, Spain & EUREGHA, European Regional and Local Health Association, Brussels, Belgium
117. Policlínica Geral do Rio de Janeiro, Rio de Janeiro – Brasil
118. Department of Medicine, Surgery and Dentistry "Scuola Medica Salernitana", University of Salerno, Salerno, Italy.
119. Peercode BV, Geldermalsen,The Netherlands.
120. Social workers oordinator, Sorrento, Italy.
121. Federal University of the State of Rio de Janeiro, School of Medicine and Surgery, Rio de Janeiro, Brazil
122. Allergology and Immunology Discipline, "Iuliu Hatieganu" University of Medicine and Pharmacy, Cluj-Napoca, Romania.
123. Department of Medicine, Division of Clinical Immunology and Allergy, McMaster University, Hamilton, Ontario, Canada.
124. Laboratoire de Pharmacologie Respiratoire UPRES EA220, Hôpital Foch, Suresnes, Université Versailles Saint-Quentin, Université Paris Saclay, France.
125. Farmacie Dei Golfi Group, Massa Lubrense, Italy.
126. Rangueil-Larrey Hospital, Respiratory Diseases Department, Toulouse, France.
127. University Clinic of Pulmology and Allergy, Medical Faculty Skopje, Republic of Macedonia.
128. Allergologo, Mexico City, Mexico..

129. Service de Pneumo-Allergologie, Centre Hospitalo-Universitaire de Béni-Messous, Algiers, Algeria.
130. Clinic of infectious, chest diseases, dermatology and allergology, Vilnius University, Vilnius, Lithuania.
131. Allergy and Clinical Immunology National Heart and Lung Institute, Imperial College London, UK.
132. Guy's and st Thomas' NHS Trust, Kings College London, UK.
133. Section of Allergy and Immunology, Saint Louis University School of Medicine, Saint Louis, Missouri, USA.

134. Pediatric Allergy and Immunology Unit, Children's Hospital, Ain Shams University, Cairo, Egypt.
135. Department of Computing Science, Umeå University, Sweden and Four Computing Oy, Finland.

136. Clinic of Children's Diseases, Faculty of Medicine, Vilnius University, Vilnius, Lithuania.
137. University of São Paulo Medical School, Sao Paulo, Brazil

138. Andalusian Agency for Healthcare Quality, Seville, Spain.
139. Global Allergy and Asthma Platform GAAPP, Vienna, Austria.
140. Division of Allergy, Department of Pediatric Medicine - The Bambino Gesù Children's Research Hospital Holy see, Rome, Italy.
141. Department of Otorhinolaryngology, Academic Medical Centers, Amsterdam, the Netherlands.
142. CINTESIS, Center for Research in Health Technologies and Information Systems, Faculdade de Medicina da Universidade do Porto, Porto, Portugal and MEDIDA, Lda, Porto, Portugal
143. Allergist, Reims, France.
144. Hospital General Regional 1 "Dr Carlos Mc Gregor Sanchez Navarro" IMSS, Mexico City, Mexico.

145. Regional hospital of ISSSTE, Puebla, Mexico.
146. National Center for Disease Control and Public Health of Georgia, Tbilisi, Georgia.
147. Allergologo, Guadalajara, Mexico.

148. Allergy Clinic, National Institute of Respiratory Diseases, Mexico City, Mexico.
149. Department of Pulmonary Diseases, Istanbul University-Cerrahpasa, Cerrahpasa Faculty of Medicine, Istambul,Turkey.
150. Allergology unit, UHATEM "NIPirogov", Sofia, Bulgaria.
151. Medical University, Faculty of Public Health, Sofia, Bulgaria.
152. Allergy and Immunology Division, Clinica Ricardo Palma, Lima, Peru.
153. Department of Internal Medicine, section of Allergology, Erasmus MC, Rotterdam, The Netherlands.
154. Allergy & Asthma Unit, Hospital San Bernardo Salta, Argentina.
155. Allergy Clinic, Hospital Regional del ISSSTE 'Lic. López Mateos', Mexico City, Mexico.
156. Head and Professor, Centro Regional de Excelencia CONACYT y WAO en Alergia, Asma e Inmunologia, Hospital Universitario, Universidad Autónoma de Nuevo León , Monterrey NL, Mexico.

157. Center of Allergy and Immunology, Georgian Association of Allergology and Clinical Immunology, Tbilisi, Georgia.
158. Latvian Association of Allergists, Center of Tuberculosis and Lung Diseases, Riga, Latvia.
159. Federal District Base Hospital Institute, Brasília, Brazil.
160. Institute of Health Policy and Management iBMG, Erasmus University, Rotterdam, The Netherlands.
161. University Hospital Olomouc – National eHealth Centre, Czech Republic.
162. Immunology and Allergy Division, Clinical Hospital, University of Chile, Santiago, Chile.
163. Skin and Allergy Hospital, Helsinki University Hospital, University of Helsinki, Helsinki, Finland.
164. Centich : centre d'expertise national des technologies de l'information et de la communication pour l'autonomie, Gérontopôle autonomie longévité des Pays de la Loire, Conseil régional des Pays de la Loire, Centre d'expertise Partenariat Européen d'Innovation pour un vieillissement actif et en bonne santé, Nantes, France.
165. Autonomous University of Baja California, Ensenada, Baja California, Mexico.

166. Department of Paediatrics and Child Health, University College Cork, Cork, Ireland.
167. Hospital General Regional 1 "Dr. Carlos MacGregor Sánchez Navarro" IMSS, Mexico City, Mexico.

168. Université Paris-Sud; Service de Pneumologie, Hôpital Bicêtre; Inserm UMR_S999, Le Kremlin Bicêtre, France.
169. Dipartimento di medicina, chirurgia e odontoiatria, università di Salerno, Italy.

170. Division for Health Innovation, Campania Region and Federico II University Hospital Naples (R&D and DISMET) Naples, Italy.
171. Servicio de Alergia e Immunologia, Clinica Santa Isabel, Buenos Aires, Argentina.
172. President, Libra Foundation, Buenos Aires, Argentina.
173. Medical University of Gdańsk, Department of Allergology, Gdansk, Poland.
174. Airway Disease Infection Section, National Heart and Lung Institute, Imperial College; MRC & Asthma UK Centre in Allergic Mechanisms of Asthma, London, UK.
175. Dept of Respiratory Medicine, Ghent University Hospital, Ghent, Belgium.
176. Hallym University College of Medicine, Hallym University Sacred Heart Hospital, Gyeonggi-do, South Korea.
177. Department of Clinical Immunology, Wrocław Medical University, Poland.
178. Ukrainina Medical Stomatological Academy, Poltava, Ukraine.
179. Pediatric Allergy and Asthma Unit, Hacettepe University School of Medicine, Ankara, Turkey.

180. Hacettepe University, School of Medicine, Department of Chest Diseases, Immunology and Allergy Division, Ankara, Turkey.
181. Allergy Centre, Tampere University Hospital, Tampere, Finland.
182. First Department of Family Medicine, Medical University of Lodz, Poland.
183. Institute of Social Medicine, Epidemiology and Health Economics, Charité - Universitätsmedizin Berlin, Berlin, and Institute for Clinical Epidemiology and Biometry, University of Wuerzburg, Germany.
184. Department of Medicine, McMaster University, Health Sciences Centre 3V47, West, Hamilton, Ontario, Canada.
185. National Research Center, Institute of Immunology, Federal Medicobiological Agency, Laboratory of Molecular immunology, Moscow, Russian Federation.
186. GARD Chairman, Geneva, Switzerland.
187. Allergy & Asthma Center Westend, Berlin, Germany.
188. Center for Rhinology and Allergology, Wiesbaden, Germany.
189. Department of Immunology and Allergy, Healthy Ageing Research Center, Medical University of Lodz, Lodz, Poland.
190. Children's Hospital and University of Helsinki, Finland.
191. Department of Clinical Science and Education, Södersjukhuset, Karolinska Institutet, Stockholm and Sach´s Children and Youth Hospital, Södersjukhuset, Stockholm, Sweden.
192. Faculty of Medicine, Vilnius University, Vilnius, Lithuania.
193. Department of Prevention of Envinronmental Hazards and Allergology, Medical University of Warsaw, Poland.
194. Center of Excellence in Asthma and Allergy, Médica Sur Clinical Foundation and Hospital, México City, Mexico.
195. Presidente CMMC, Milano, Italy.
196. Head of the Allergy Department of Pedro de Elizalde Children's Hospital, Buenos Aires, Argentina.
197. University of Medicine and Pharmacy, Hochiminh City, Vietnam.
198. Federal University of Bahia, Brazil.
199. Sifmed, Milano, Italy.
200. State Key Laboratory of Respiratory Diseases, Guangzhou Institute of Respiratory Disease, the First Affiliated Hospital of Guangzhou Medical University, Guangzhou, China.
201. Departments of Internal Medicine and Pediatrics (Divisions of Allergy and Immunology), University of Tennessee College of Medicine, Germantown, TN, USA.
202. Scottish Centre for Respiratory Research, Cardiovascular & Diabetes Medicine, Medical Research Institute, Ninewells Hospital, University of Dundee, UK.
203. Oslo University Hospital, Department of Paediatrics, Oslo, and University of Oslo, Faculty of Medicine, Institute of Clinical Medicine, Oslo, Norway.
204. Department of Pulmonary Medicine, CHU Sart-Tilman, and GIGA I3 research group, Liege, Belgium.
205. Faculty of Health Sciences and CICS – UBI, Health Sciences Research Centre, University of Beira Interior, Covilhã, Portugal.
206. Department of Philosophical, Methodological and Instrumental Disciplines, CUCS, University of Guadalajara, Guadalajara, Mexico.
207. Department of Pulmonary Medicine, Rashid Hospital, Dubai, UAE.
208. Biomax Informatics AG, Munich, Germany.
209. Director General for Health and Social Care, Scottish Government, Edinburgh, UK.
210. Department of Respiratory Medicine, University of Bratislava, Bratislava, Slovakia.
211. Coimbra Institute for Clinical and Biomedical Research (iCBR), Faculty of Medicine, University of Coimbra, Portugal; Ageing@Coimbra EIP-AHA Reference Site, Coimbra, Portugal.

212. Medical center Iskar Ltd Sofia, Bulgaria.
213. Department of Medicine (RCSI), Bon Secours Hospital, Glasnevin, Dublin, Ireland.
214. Kronikgune, International Centre of Excellence in Chronicity Research Barakaldo, Bizkaia, Spain
215. Division of Clinical Immunology and Allergy, Laboratory of Behavioral Immunology Research, The University of Mississippi Medical Center, Jackson, Mississippi, USA.
216. Tobacco Control Research Centre;Iranian Anti Tobacco Association, Tehran, Iran.
217. Argentine Association of Allergy and Clinical Immunology, Buenos Aires, Argentina.
218. Hospital de Especialidades, Centro Medico Nacional Siglo XXI, Mexico City, Mexico

219. University of Southeast Bahia, Brazil.
220. Allergie-Centrum-Charité at the Department of Dermatology and Allergy, Charité - Universitätsmedizin Berlin, Germany
221. Maputo Central Hospital, Department of Paediatrics, Maputo, Mozambique.
222. Allergologo, Veracruz, Mexico

223. Sachs' Children and Youth Hospital, Södersjukhuset, Stockholm and Institute of Environmental Medicine, Karolinska Institutet, Stockholm, Sweden.
224. Allergy and Asthma Medical Group and Research Center, San Diego, California, USA.

225. CIRFF, Federico II University, Naples, Italy.
226. Department of Physiology, CHRU, University Montpellier, Vice President for Research, PhyMedExp, INSERM U1046, CNRS UMR 9214, France.
227. Croatian Pulmonary Society.
228. National Institute of Pneumology M Nasta, Bucharest, Romania.
229. Clinic for Pulmonary Diseases, Clinical Center of Serbia, Faculty of Medicine, University of Belgrade, Serbian Association for Asthma and COPD, Belgrade, Serbia.
230. Regione Piemonte, Torino, Italy.
231. Col Jardines de Sta Monica, Tlalnepantla, Mexico.

232. National Center for Research in Chronic Respiratory Diseases, Tishreen University School of Medicine, Latakia, Syria.
233. Department of Public health and health products, Paris Descartes University-Sorbonne Paris Cité, EA 4064 and Paris Municipal Department of social action, childhood, and health, Paris, France .
234. Paris municipal Department of social action, childhood, and health, Paris, France.
235. Lead Respiratory Physician Mater Dei Hospital Malta, Academic Head of Dept and Professor of Medicine University of Malta, Deputy Dean Faculty of Medicine and Surgery University of Medicine, La Valette, Malta.
236. Department of Medical Sciences, Allergy and Clinical Immunology Unit, University of Torino & Mauriziano Hospital, Torino, Italy.
237. Instituto de Prevision Social IPS HC, Socia de la SPAAI, Tesorera de la SLAAI, Asuncion, Paraguay.
238. Allergy Center, CUF Descobertas Hospital, Lisbon, Portugal.
239. Universidade de São Paulo, São Paulo, Brazil.
240. Institute of Medical Statistics, and Computational Biology, Medical Faculty, University of Cologne, Germany and CRI-Clinical Research International-Ltd, Hamburg, Germany.
241. General Pathology Institute, Faculty of Medicine, University of Coimbra, Portugal; Ageing@Coimbra EIP-AHA Reference Site, Coimbra, Portugal.
242. Federal University of Bahia, Brazil.
243. Rhinology Unit & Smell Clinic, ENT Department, Hospital Clínic; Clinical & Experimental Respiratory Immunoallergy, IDIBAPS, CIBERES, University of Barcelona, Spain.
244. Danish Commitee for Health Education, Copenhagen East, Denmark.
245. Food Allergy Referral Centre Veneto Region, Department of Women and Child Health, Padua General University Hospital, Padua, Italy.
246. Director, Medical Communications Consultant, MedScript Ltd, Dundalk, Co Louth, Ireland and New Zealand, and Honorary Research Fellow, OPC, Cambridge, UK.
247. Johns Hopkins School of Medicine, Baltimore, Maryland, USA.
248. General Manager of COFASER - Pharmacy Services Consortium, Salerno, Italy.

249. Scientific Centre of Children's Health under the MoH, Moscow, Russian National Research Medical University named Pirogov, Moscow, Russia.
250. Director of Center of Allergy, Immunology and Respiratory Diseases, Santa Fe, Argentina Center for Allergy and Immunology, Santa Fe, Argentina.
251. Dept of Otorhinolaryngology, Medical University of Vienna, AKH, Vienna, Austria.
252. Hospital of the Hospitaller Brothers in Buda, Budapest, Hungary.
253. Die Hautambulanz and Rothhaar study center, Berlin, Germany.
254. Neumología y Alergología Infantil, Hospital La Fe, Valencia, Spain.
255. Center for Health Technology and Services Research - CINTESIS and Department of Internal Medicine, Centro Hospitalar Sao Joao, Porto, Portugal.
256. Caisse d'assurance retraite et de la santé au travail du Languedoc-Roussillon (CARSAT-LR), Montpellier, France.
257. Director of Department of Pharmacy of University of Naples Federico II, Naples, Italy.
258. ENT Department, University Hospital of Kinshasa, Kinshasa, Congo.
259. Department of Allergy, Immunology and Respiratory Medicine, Alfred Hospital and Central Clinical School, Monash University, Melbourne, Victoria, Australia; Department of Immunology, Monash University, Melbourne, Victoria, Australia.
260. Medical center "Research expert", Varna, Bulgaria.
261. National Hospital Organization, Tokyo National Hospital, Tokyo, Japan.
262. Dept of Otorhinolaryngology, Chiba University Hospital, Chiba, Japan.
263. Dept of Otolaryngology, Nippon Medical School, Tokyo, Japan.
264. Allergologo, Jalisco, Guadalajara, Mexico.

265. Centre Hospitalier Universitaire Pédiatrique Charles de Gaulle, Ouagadougou, Burkina Faso.
266. Dept of Comparative Medicine; Messerli Research Institute of the University of Veterinary Medicine and Medical University, Vienna, Austria.

267. Department of Immunology and Allergology, Faculty of Medicine and Faculty Hospital in Pilsen, Charles University in Prague, Pilsen, Czech Republic.
268. Division of Infection, Immunity & Respiratory Medicine, Royal Manchester Children's Hospital, University of Manchester, Manchester, UK, and Allergy Department, 2nd Pediatric Clinic, Athens General Children's Hospital "P&A Kyriakou," University of Athens, Athens, Greece.
269. Department of Allergy and Clinical Immunology, Ajou University School of Medicine, Suwon, South Korea.
270. Respiratory Medicine, Department of Medical Sciences, University of Ferrara, Ferrara, Italy.
271. Allergy and Respiratory Diseases, Ospedale Policlino San Martino -University of Genoa, Italy.
272. Farmacias Holon, Lisbon, Portugal.
273. Department of Pediatrics, Nippon Medical School, Tokyo, Japan.
274. University of Southern Denmark, Kolding, Denmark.
275. Université Grenoble Alpes, Laboratoire HP2, Grenoble, INSERM, U1042 and CHU de Grenoble, France.
276. Allergy Unit, CUF-Porto Hospital and Institute; Center for Research in Health Technologies and information systems CINTESIS, Universidade do Porto, Portugal.
277. Sociologist, municipality area n33, Sorrento, Italy.
278. Center for Rhinology and Allergology, Wiesbaden, Germany.
279. Department of Otorhinolaryngology, Head and Neck Surgery, Universitätsmedizin Mannheim, Medical Faculty Mannheim, Heidelberg University, Mannheim, Germany.
280. Centre for empowering people and communites, Dublin, UK.
281. Conseil Général de l'Economie Ministère de l'Economie, de l'Industrie et du Numérique, Paris, France.
282. Société de Pneumologie de Langue Française, Espace francophone de Pneumologie, Paris, France.
283. Département de pédiatrie, CHU de Grenoble, Grenoble France.
284. Medical School, University of Cyprus, Nicosia, Cyprus.
285. Children’s Hospital Srebrnjak, Zagreb, School of Medicine, University J.J. Strossmayer, Osijek, Croatia.
286. Karl Landsteiner Institute for Clinical and Experimental Pneumology, Hietzing Hospital, Vienna, Austria.
287. University Hospital 'Sv. Ivan Rilski'", Sofia, Bulgaria.
288. Allergy Diagnostic and Clinical Research Unit, University of Cape Town Lung Institute, Cape Town, South Africa.
289. Vice-Presidente of IML, Milano, Italy.
290. Centre of Academic Primary Care, Division of Applied Health Sciences, University of Aberdeen, Aberdeen, United Kingdom ; Observational and Pragmatic Research Institute, Singapore, Singapore.
291. Department of Otorhinolaryngology University of Crete School of Medicine, Heraklion, Greece.
292. European Forum for Research and Education in Allergy and Airway Diseases (EUFOREA), Brussels, Belgium.
293. Allergologo, cancun quintana roo, Mexico.

294. LungenClinic Grosshansdorf, Airway Research Center North, Member of the German Center for Lung Research (DZL), Grosshansdorf, Germany Department of Medicine, Christian Albrechts University, Airway Research Center North, Member of the German Center for Lung Research (DZL), Kiel, Germany.
295. Department of Nephrology and Endocrinology, Karolinska University Hospital, Stockholm, Sweden.
296. Farmácia São Paio, Vila Nova de Gaia, Porto, Portugal.
297. St Vincent’s Hospital and University of Sydney, Sydney, New South Wales, Australia.
298. Allergologo, Mexico City, Mexico

299. Serviço de Pneumologia-Hosp das Clinicas UFPE-EBSERH, Recife, Brazil.
300. Universidade Federal de São Paulo, São Paulo, Brazil.
301. Centre of Pneumology, Coimbra University Hospital, Portugal.
302. Polibienestar Research Institute, University of Valencia, Valencia, Spain.
303. Pediatric Allergy and Clinical Immunology, Hospital Angeles Pedregal, Mexico City, Mexico.
304. Getafe University Hospital Department of Geriatrics, Madrid, Spain.
305. Association Asthme et Allergie, Paris, France.
306. Universidade Federal do Rio de Janeiro, Rio de Janeiro, Brazil.
307. Primary Care Respiratory Research Unit Institutode Investigación Sanitaria de Palma IdisPa, Palma de Mallorca, Spain.
308. Allergy Unit, Presidio Columbus, Rome, Catholic University of Sacred Heart, Rome and IRCCS Oasi Maria SS, Troina, Italy.
309. Hospital General, Mexico City, Mexico.

310. Regione Piemonte, Torino, Italy.
311. Medical University of Graz, Department of Internal Medicine, Graz, Austria.
312. Serviço de Imunoalergologia Hospital da Luz, Lisboa, Portugal.
313. Hospital de Clinicas, University of Parana, Brazil.
314. Division of Allergy Asthma and Clinical Immunology, Emek Medical Center, Afula, Israel.
315. Honorary Clinical Research Fellow, Allergy and Respiratory Research Group, The University of Edinburgh, Edinburgh, UK.

316. Showa University School of Medicine, Tokyo, Japan.
317. Association of Finnish Pharmacies, Helsinki, Finland.
318. Allergy and Clinical Immunology Department, Centro Médico-Docente la, Trinidad and Clínica El Avila, Caracas, Venezuela.
319. Faculty of Medicine, Autnonous University of Madrid, Spain.
320. The Royal National TNE Hospital, University College London, UK.
321. DIBIMIS, University of Palermo, Italy.
322. Allergy Unit, Department of Dermatology, University Hospital of Zurich, Zürich, Switzerland.
323. Asthma Reference Center, Escola Superior de Ciencias da Santa Casa de Misericordia de Vitoria - Esperito Santo, Brazil.
324. The Usher Institute of Population Health Sciences and Informatics, The University of Edinburgh, Edinburgh, UK.
325. Department of Pediatrics & Child Health, Department of Immunology, Faculty of Medicine, University of Manitoba, Winnipeg, Manitoba, Canada.
326. INSERM, Université Grenoble Alpes, IAB, U 1209, Team of Environmental Epidemiology applied to Reproduction and Respiratory Health, Université Joseph Fourier, Grenoble, France.
327. Sociedad Paraguaya de Alergia Asma e Inmunologı´a, Paraguay.
328. Division of Allergy, Clinical Immunology and Rheumatology, Department of Pediatrics, Federal University of São Paulo, São Paulo, Brazil.
329. European Health Futures Forum (EHFF), Dromahair, Ireland.
330. ENT, Aachen, Germany.
331. Kyrgyzstan National Centre of Cardiology and Internal medicine, Euro-Asian respiratory Society, Bishkek, Kyrgyzstan.
332. University Hospital Olomouc, Czech Republic.
333. Department of Paediatric and Adolescent medicine, University Hospital of North Norway, Tromsø, Paediatric Research Group, Deptarment of Clinical Medicine, Faculty of Health Sciences, UiT The Arctic University of Norway, Tromsø, Norway.
334. Presidente, IML (Lombardy Medical Initiative), Bergamo, Italy.
335. Pulmonary Division, Heart Institute (InCor), Hospital da Clinicas da Faculdade de Medicina da Universidade de Sao Paulo, Sao Paulo, Brazil.
336. Public Health Institute of Vilnius University, Vilnius, Lithuania.
337. Universidade Federal do Estado do Rio de Janeiro, Rio de Janeiro, Brazil.
338. RNSA (Réseau National de Surveillance Aérobiologique), Brussieu, France.
339. The Hospital for Sick Children, Dalla Lana School of Public Health, University of Toronto, Canada.
340. Imunoalergologia, Centro Hospitalar Universitário de Coimbra and Faculty of Medicine, University of Coimbra, Portugal.
341. Department of ENT, Medical University of Graz, Austria.
342. Campania Region, Division on Pharmacy and devices policy, Naples, Italy.
343. Department of Respiratory Medicine, Hvidovre Hospital & University of Copenhagen, Denmark.
344. Universidade Federal dos Pampas, Uruguaiana, Brazil.
345. Division of Immunopathology, Department of Pathophysiology and Allergy Research, Center for Pathophysiology, Infectiology and Immunology, Medical University of Vienna, Vienna, Austria.
346. Pneumology and Allergy Department CIBERES and Clinical & Experimental Respiratory Immunoallergy, IDIBAPS, University of Barcelona, Spain.
347. Vilnius University Institute of Clinical Medicine, Clinic of Children's Diseases, and Institute of Health Sciences, Department of Public Health, Vilnius, Lithuania; European Academy of Paediatrics (EAP/UEMS-SP), Brussels, Belgium.
348. Department of Lung Diseases and Clinical Immunology Allergology, University of Turku and Terveystalo allergy clinic, Turku, Finland.
349. PELyon; HESPER 7425, Health Services and Performance Resarch - Université Claude Bernard Lyon, France.
350. Immunology and Allergy Unit, Department of Medicine Solna, Karolinska Institutet and University Hospital, Stockholm.
351. Department of Chest Medicine, Centre Hospitalier Universitaire UCL Namur, Université Catholique de Louvain, Yvoir, Belgium.
352. University of Bari Medical School, Unit of Geriatric Immunoallergology, Bari, Italy.
353. Pulmonary Unit, Department of Medical Specialties, Arcispedale SMaria Nuova/IRCCS, AUSL di Reggio Emilia, Italy.
354. FILHA, Finnish Lung Association, Helsinki, Finland.
355. Pulmonary Environmental Epidemiology Unit, CNR Institute of Clinical Physiology, Pisa, Italy ; and CNR Institute of Biomedicine and Molecular Immunology "A Monroy", Palermo, Italy.
356. Medical University, Plovdiv, Bulgaria, Department of Otorhinolaryngology, Plovdiv, Bulgaria.
357. Sotiria Hospital, Athens, Greece.
358. Dept of Otorhinolaryngology, Universitätsklinikum Düsseldorf, Germany.
359. Asthma UK, Mansell street, London, UK.
360. Nova Southeastern University, Fort Lauderdale, Florida, USA.

361. Department of Otolaryngology, Yong Loo Lin School of Medicine, National University of Singapore, Singapore, Singapore.
362. Department of Medicine, Clinical Immunology and Allergy, McMaster University, Hamilton, Ontario, Canada.
363. Division of Immunodermatology and Allergy Research, Department of Dermatology and Allergy, Hannover Medical School, Hannover, Germany.
364. Department of Medicine Solna, Immunology and Allergy Unit, Karolinska Institutet and Department of ENT diseases, Karolinska University Hospital, Stockholm, Sweden.
365. Eshelman School of Pharmacy, University of North Carolina, Chapel Hill, NC, USA.
366. International Primary Care Respiratory Group IPCRG, Aberdeen, Scotland.
367. Bradford Institute for Health Research, Bradford Royal Infirmary, Bradford, UK.
368. Allergologyst - Medical College of Medical Faculty, Thracian University, Stara Zagora, Bulgaria.
369. Department of Research, Olmsted Medical Center, Rochester, Minnesota, USA.
370. Cyprus International Institute for Environmental & Public Health in Association with Harvard School of Public Health, Cyprus University of Technology, Limassol, Cyprus; Department of Pediatrics, Hospital "Archbishop Makarios III", Nicosia, Cyprus.
371. Celal Bayar University Department of Pulmonology, Manisa, Turkey.
372. The Allergy and Asthma Institute, Islamabad, Pakistan.
373. Department of Paediatrics and Child Health, Red Cross Children's Hospital, and MRC Unit on Child & Adolescent Health, University of Cape Town, Cape Town, South Africa.
374. Department of Otolaryngology Head and Neck Surgery, Beijing TongRen Hospital and Beijing Institute of Otolaryngology, Beijing, China.
375. Universidad Católica de Córdoba, Córdoba, Argentina.
376. University Clinic of Respiratory and Allergic Diseases, Golnik, Slovenia.
377. Gesundheitsregion KölnBonn - HRCB Projekt GmbH, Kohln, Germany.
378. Akershus University Hospital, Department of Otorhinolaryngology, Akershus, Norway.
379. Chief of Staff, the Northern Health Science Alliance (NHSA) and Director and Founder of Northern Health Matters Ltd, Manchester, UK.
380. President of Kazakhstan Association of Allergology and Clinical Immunology, Department of Allergology and Clinical Immunology of the Kazakh National Medical University, Kazakhstan.
381. Division of Respiratory and Allergic Diseases,Hospital 'A Cardarelli', University of Naples Federico II, Naples, Italy.
